# Natural variations of chlorophyll fluorescence and ion transporter genes influenced the differential response of *japonica* rice germplasm with different salt tolerances

**DOI:** 10.3389/fpls.2023.1095929

**Published:** 2023-03-17

**Authors:** Jiawei Song, Hui Yang, Chengbin Qiao, Chunyan Zhu, Tianliang Bai, Huaidong Du, Shuaiguo Ma, Na Wang, Chengke Luo, Yinxia Zhang, Tianli Ma, Peifu Li, Lei Tian

**Affiliations:** ^1^ School of Agriculture, Ningxia University, Yinchuan, China; ^2^ Key Laboratory of Modern Molecular Breeding for Dominant and Special Crops in Ningxia, Ningxia University, Yinchuan, China; ^3^ Agricultural College, Tarim University, Alar, China

**Keywords:** *japonica* rice germplasm, salt stress, chlorophyll fluorescence, Na^+^/K^+^ homeostasis, haplotype analysis

## Abstract

Soil salinity seriously restricts rice growth, development, and production globally. Chlorophyll fluorescence and ion content reflect the level of injury and resistance of rice under salt stress. To understand the differences in the response mechanisms of *japonica* rice with varying degrees of salt tolerance, we analyzed the chlorophyll fluorescence characteristics and ion homeostasis of 12 *japonica* rice germplasm accessions by comprehensive evaluation of phenotype, haplotype, and expression of salt tolerance-related genes. The results revealed that salt-sensitive accessions were rapidly affected by the damage due to salinity. Salt tolerance score (STS) and relative chlorophyll relative content (RSPAD) were extremely significantly reduced (p<0.01), and chlorophyll fluorescence and ion homeostasis were influenced by various degrees under salt stress. The STS, RSPAD, and five chlorophyll fluorescence parameters of salt-tolerant accessions (STA) were significantly higher than that of salt-sensitive accessions (SSA). Principal component analysis (PCA) with 13 indices suggested three principal components (PCs), with a cumulative contribution rate of 90.254%, which were used to screen Huangluo (typical salt-tolerant germplasm) and Shanfuliya (typical salt-sensitive germplasm) based on the comprehensive evaluation *D*-value (*D_CI_*). The expression characteristics of chlorophyll fluorescence genes (*OsABCI7* and *OsHCF222*) and ion transporter protein genes (*OsHKT1;5*, *OsHKT2;1*, *OsHAK21*, *OsAKT2*, *OsNHX1*, and *OsSOS1*) were analyzed. The expressions of these genes were higher in Huangluo than in Shanfuliya under salt stress. Haplotype analysis revealed four key variations associated with salt tolerance, including an SNP (+1605 bp) within *OsABCI7* exon, an SSR (−1231 bp) within *OsHAK21* promoter, an indel site at *OsNHX1* promoter (−822 bp), and an SNP (−1866 bp) within *OsAKT2* promoter. Variation in OsABCI7 protein structure and differential expression of these three ion-transporter genes may contribute to the differential response of *japonica* rice to salt stress.

## Introduction

Soil salinization is one of the major limiting factors for crop growth and production (Wei et al., 2021). Saline soils are widely distributed globally in 100 countries on 6 continents, with a total area of 1.173 × 10^7^ km^2^ (Hassani et al., 2021). Rice (*Oryza sativa* L.), a major food crop in Asia, is a salt-sensitive crop. Particularly, it is sensitive to salt stress during the seedling and reproductive stages (Ganie et al., 2019). Generally, damage to rice due to salt stress mainly occurs *via* osmotic stress, membrane lipid peroxidation, and ion toxicity (Lee et al., 2013; Shen et al., 2015). Salt stress increases Na^+^ content in rice cells and reduces K^+^ uptake under ion competition, resulting in increased Na^+^/K^+^ and disruption of ion balance eventually leading to cell death under the dual action of osmotic stress and ion poisoning (Feng et al., 2019).

Maintaining the dynamic balance of Na^+^ and K^+^ is an important means for plants to cope with salt stress, mainly through long-distance transport of ions in the xylem and phloem and accumulation and compartmentalization of ions (Liu et al., 2022). Ion-transport proteins are the key factors in the maintenance of Na^+^ and K^+^ homeostasis in plants under salt stress (Jabnoune et al., 2009). In rice, several transporter protein families, mainly HKT, HAK, AKT, NHX, and SOS, regulate ion homeostasis. The high-affinity K^+^ transporter (HKT) family in rice has two subfamilies. A member of the subfamily I, namely, *OsHKT1;5* (*SKC1*), transports Na^+^ from rice shoot to root *via* xylem unloading to reduce Na^+^ accumulation in the shoot under salt stress (Ren et al., 2005). A member of the subfamily II, namely, *OsHKT2;1* (*OsHKT1*), maintains cytoplasmic Na^+^/K^+^ homeostasis by selectively transporting Na^+^ or Na^+^ and K^+^ under the regulation of the clock component OsPRR73 (Jabnoune et al., 2009; Wei et al., 2021). *OsHAK21* (*qSE3*), a member of the HAK/KUP/KT transporter family, regulates salt tolerance at the germination and seedling establishment stages of rice by regulating K^+^ and Na^+^ uptake, increasing abscisic acid (ABA) biosynthesis, and activating ABA signaling responses (He et al., 2019). Shaker K^+^ channel protein, namely, OsAKT2, plays an important role in K^+^ loading and redistribution from the phloem and maintains cellular Na^+^/K^+^ homeostasis by regulating the K^+^ redistribution process (Tian et al., 2021). OsNHX1, a vesicular membrane NHX-type antiporter, reverses cation transport into the vesicles by ion compartmentalization under salt stress to maintain cellular ion homeostasis and osmotic potential (Fukuda et al., 2004; Liu et al., 2010; Fukuda et al., 2011; Ma et al., 2016; Bassil et al., 2018; Wu et al., 2018). Rice plasma membrane Na^+^/H^+^ exchange protein, namely, salt overly sensitive 1 (OsSOS1), which is activated by the protein kinase complex OsCIPK24-OsCBL4, maintains salt tolerance in rice in a specific manner by Na^+^ efflux (Martínez-Atienza et al., 2007; El Mahi et al., 2019).

Chlorophyll fluorescence can accurately reflect the process of light energy uptake and conversion by plants and effect of salt stress on the photosynthetic system of plants. In rice leaves, salt stress disrupts the balance of production and scavenging of reactive oxygen species (ROS) on the thylakoid membrane. This causes oxidative reactions that damage the thylakoid membrane, reduce the regulation of excitation energy partitioning between photosystems PSI and II, reduce photosynthetic efficiency, and alter fluorescence properties (Faseela et al., 2020; He et al., 2020). Theerawitaya et al. explored the physiological characteristics of rice varieties Pokkali (salt tolerant) and IR29 (salt sensitive) under salt stress and observed that the maximal fluorescence (Fm), maximum quantum efficiency of PSII (Fv/Fm), and net photosynthetic rate (Pn) were significantly higher in Pokkali than in IR29 after salt stress (Theerawitaya et al., 2020). Moreover, Singh et al. confirmed that Fv/Fm of salt-sensitive rice varieties decreased significantly with increased duration of salt stress, whereas no significant changes were observed in salt-tolerant varieties (Singh and Sarkar, 2014). He et al. reported that OsHCF222, a high chlorophyll fluorescent protein localized in the endoplasmic reticulum and chloroplasts, is a key factor regulating chlorophyll fluorescence properties of rice. This protein forms a complex by binding to its interacting protein OsABCI7, which regulates cellular ROS homeostasis to maintain the stability of the thylakoid membrane (He et al., 2020). Zhu et al. reported that the differential expression of *OsHCF222* and *OsABCI7* directly affected the changes in chlorophyll fluorescence parameters of *japonica* rice germplasm with different salt tolerances and may be involved in the regulation of salt tolerance in rice seedlings (Zhu et al., 2022).

In recent years, many studies have reported the regulation of salt tolerance in rice by ion homeostasis and chlorophyll fluorescence, focusing on the phenotype or gene expression. However, only few studies have comprehensively evaluated salt tolerance in rice by combined analysis of phenotype, haplotype, and gene expression. It is largely unclear how these three aspects interact and regulate each other, and the key reasons for differential responses to salt stress by salt-tolerant and salt-sensitive accessions are not studied. In this study, we aimed to provide foundational insights into these issues. Morphological indices and physiology indices associated with ion homeostasis and chlorophyll fluorescence were examined for 12 *japonica* rice germplasm accessions with different salt tolerances. The expression characteristics and haplotypes of ion-transporter proteins and chlorophyll fluorescence-related genes were analyzed. This study provided an important reference for studying molecular mechanisms of salt tolerance in rice.

## Materials and methods

### Plant materials, growth conditions, and salt stress treatment

In a previous study, we comprehensively evaluated 165 *japonica* rice germplasm accessions for salt tolerance at the seedling stage (Ma et al., 2020). According to *D_ST_* and salt tolerance score (STS) (Wang et al., 2015), six salt-tolerant accessions (STA) and six salt-sensitive accessions (SSA) were selected as the experimental material (**Table 1**).

**Table 1:** Origins and names of twelve *japonica* rice germplasm accessions with different salt tolerances, their salt tolerance type, value *D_ST_*, and STS.

No.	Name	Origin	Salt tolerance	*D_ST_*	STS
1	Bertone	Portugal	salt-tolerant	0.810	7.5
2	Agostono	Italy	salt-tolerant	0.716	7.2
3	Huangluo	Russia	salt-tolerant	0.681	6.6
4	Yangbiguangkeludao	Yunnan, China	salt-tolerant	0.627	6.3
5	Cigalon	France	salt-tolerant	0.621	6.5
6	Banat 2951	Australia	salt-tolerant	0.612	6.5
7	Shanfuliya	Guinea	salt-sensitive	0.545	1.4
8	Nipponbare	Japan	salt-sensitive	0.521	2.9
9	Xiannan 22	Korea	salt-sensitive	0.497	1.7
10	Koshihikari	Japan	salt-sensitive	0.312	1.8
11	Jianan 8	Taiwan, China	salt-sensitive	0.261	2.2
12	Banat 725	Australia	salt-sensitive	0.252	2.6

*D_ST_: D* value of comprehensive evaluation on salt tolerance of *japonica* rice germplasm at the seedling stage, based on 12 morphological indices.

The experiments were conducted in mid-to-late July 2021 in the solar greenhouse of Ningxia University. After breaking the dormancy, seeds of the 12 *japonica* rice germplasm accessions were surface-sterilized using 5% sodium hypochloride for 30 min, washed with distilled water for three times, and placed in black seedling trays with two layers of filter paper for germination. After 14 days of incubation in the full-strength Yoshida’s solution, neat, uniform, and well-grown seedlings were selected and transferred into 96-well PCR plates with 1/3 of the bottom cut off and placed in plastic boxes (66 cm × 41 cm × 16 cm) with 15 L of 1× Yoshida’s solution. Two treatments were set as follows: salt stress treatment (SST; 1× Yoshida’s solution with 125 mmol L^−1^ NaCl) and control treatment (CK; 1× Yoshida’s solution), with three replicates of each treatment and 24 plants per replicate. The 1× Yoshida’s solution was replaced every 2 days, and the pH was maintained at 5.0–5.5 by adding HCl.

### Phenotype determination

Overall, 12 plants per replicate were randomly selected from each germplasm accession during 0–10 days of salt stress to measure STS and relative chlorophyll relative content (RSPAD) according to the method described by Tian et al. (2014) and Tian et al. (2011).

The chlorophyll fluorescence parameters, namely, Fm, photosynthetic electron transfer rate (ETR), yield (Y), Fv/Fm, and non-photochemical quenching coefficient (NPQ) were determined as the key indices associated with salt tolerance as per the methods described by Zhu et al. (2022).

For cell membrane permeability (CMP) measurement, the rice leaf surface was washed three times using deionized water; appropriate amount of leaves was weighed, and the leaves were cut into 0.5-cm pieces according to the method described by Lutts et al. (1996). The leaves were divided into test tubes, and 10 mL of deionized water was added. The tubes were vacuumed for 1 h and allowed to stand for 1 h. The conductivity of the solution (L_1_) was measured using a conductivity meter. Further, it was heated in a water bath at 100° for 45 min; further, the solution conductivity (L_2_) was measured again after cooling. The electrolyte leakage was calculated as L_1_/L_2_ and expressed as %.

Antioxidant enzymes such as superoxide dismutase (SOD), peroxidase (POD), and catalase (CAT) were extracted and measured in rice leaves according to the method described by Lu et al. (2022).

Atomic absorption spectrometry (AAS) was used to determine Na^+^ and K^+^ contents in rice shoots and roots (Ren et al., 2005). After treating with NaCl, the shoots and roots were harvested, rinsed, and dried. Rice shoots or roots were weighed (0.1 g), transferred to a 50 mL tube containing 10 mL acetic acid (100 mmol L^−1^), and shaken in a water bath at 95°C for 5 h. The tubes were then centrifuged at 3000g for 5 min. After centrifugation, the supernatant was diluted 20–100 times by deionized water to obtain a final volume of 5 mL. Using this solution, the Na^+^ and K^+^ contents were detected using an atomic absorption spectrophotometer (AAS 6800 series, Shimadzu, Japan). The average values of three replicates were considered for each index.

### Quantitative real-time PCR

Total RNA and reverse-transcribed cDNA of rice seedlings were extracted according to the method described by He et al. (2020). RT-PCR was performed using a quantitative fluorescence gene amplification instrument (qTOWER3G; Jena, Germany). *OsActin* (*LOC_Os03g50885*) was used as the reference gene to analyze the expression of ion-transporter protein genes *OsHKT1;5* (*SKC1*, *LOC_Os01g20160*), *OsHKT2;1* (*OsHKT1*, *LOC_Os06g48810*), *OsAKT2* (*OsK3.1*, *LOC_Os05g35410*), *OsHAK21* (*qSE3*, *LOC_Os03g37930*), *OsNHX1* (*LOC_Os07g47100*), *OsSOS1* (*LOC_Os12g44360*), and chlorophyll fluorescence genes *OsABCI7* (*LOC_Os11g29850*) and *OsHCF222* (*LOC_Os03g30092*). Primer 5.0 was used for specific primer designing, and the PCR products were sent to the Shanghai Sangon Biological Engineering Technology Company (Shanghai, China) for sequencing. The primer sequence was given in **Supplementary Tables 1**, **2**.

### Data analysis

Excel 2019 was used to organize the experimental data. SPSS 26.0 was used to perform independent samples F-test, t-test, significant difference analysis (LSD method), and principal component analysis (PCA) for 13 salt tolerance-related indices. Correlation analysis was performed and plotted using R. Origin 2021. IBS and DNAMAN were used for gene structure drawing and sequence alignment, respectively. A comprehensive evaluation of *japonica* rice germplasm accessions under salt stress was conducted using the membership function and index weight method described by Li et al. (2020).

## Results

### STS and RSPAD analysis of *japonica* rice germplasm accessions with different salt tolerances under salt stress

**Figure 1:**
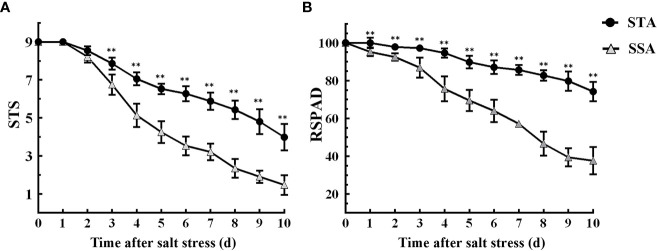
Dynamic changes in salt tolerance score (STS) and relative SPAD (RSPAD) in STA and SSA 0–10 days after salt stress. **(A)** STS, **(B)** RSPAD; **p < 0.01.

Six STA and six SSA seedlings were treated with 125 mmol L^−1^ NaCl for 10 days. The STS and RSPAD of STA and SSA gradually decreased as the duration of salt stress increased, and the decrease was more rapid in SSA (**Figure 1**). After 3 days of salt stress, the STS of STA was extremely significantly higher than that of SSA (p < 0.01), and the difference was the maximum at 6 days of salt stress (**Figure 1A**). Compared with STS, RSPAD responded more rapidly to salt stress, and the RSPAD of SSA was significantly lower than that of STA at day 1. An accelerated reduction in RSPAD of SSA was observed after 3 days of salt stress; the RSPAD of SSA at day 10 was only 40% of that at day 0. The RSPAD of STA remained at a relatively high level (always > 80%; **Figure 1B**). Based on these results, the physiological indices of STA and SSA were measured at days 3 and 6 after salt stress.

### Effects of salt stress on 13 salt-tolerance indices

The F-test and t-test results revealed that all 13 salt-tolerance indices of STA and SSA exhibited differences of variable degrees between the CK and SST groups (**Table 2**). In STA, highly significant differences were observed in terms of STS, RSPAD, shoot and root Na^+^ content (SNa^+^ and RNa^+^, respectively), shoot and root K^+^ content (SK^+^ and RK^+^, respectively), and shoot and root Na^+^/K^+^ (SNa^+^/K^+^ and RNa^+^/K^+^, respectively) at days 3 and 6 after salt stress compared with the CK group. Fm, Fv/Fm, and NPQ were significantly or highly significantly different from the CK group at day 3 after salt stress, and only Fv/Fm was significantly different from the CK group at day 6 after salt stress (p < 0.05). In SSA, highly significant differences were observed in terms of STS, RSPAD, Fm, SNa^+^, RNa^+^, SK^+^, SNa^+^/K^+^, and RNa^+^/K^+^ at days 3 and 6 after salt stress compared with the CK group. Four chlorophyll fluorescence indices, namely, Fv/Fm, Y, NPQ, and ETR, exhibited significant differences at day 3 after salt stress and highly significant difference at day 6 after salt stress compared with the CK group; RK^+^ exhibited highly significant differences compared to CK only at day 6 after salt stress. The coefficients of variation for STS, RSPAD, Fm, and Fv/Fm were small (0%–11.3%) in the CK group, whereas those for Y, ETR, SNa^+^, and RNa^+^/K^+^ were relatively large (20.1%–39.4%). Under salt stress, Y, ETR, RK^+^, and RNa^+^/K^+^ differed more in STA than in CK group, with a maximum coefficient of variation of 46.6% (RNa^+^/K^+^). SSA exhibited large variation in terms of STS, Fm, Y, NPQ, ETR, RNa^+^, SK^+^, RK^+^, SNa^+^/K^+^, and RNa^+^/K^+^ with coefficients of variation ranging from 13.19% to 68.70%. The distribution ranges of Fm, Fv/Fm, Y, and NPQ of STA did not overlap with those of SSA at 6 days after salt stress.

**Table 2:** Distribution range, coefficient of variation, F-value, and t-value of salt tolerance indices under control and salt stress in *japonica* rice germplasm accessions.

Salt tolerance indices	Treatment time	Salt-tolerant accessions				Salt-sensitive accessions			
		Range	CV%	F- Value	t-Value	Range	CV%	F- Value	t-Value
STS	0mmol/L 3d	9.00	0.00	–	15.22**	9.00	0.00	–	20.56**
	125mmol/L 3d	7.00-9.00	8.20			4.00-8.00	13.90		
	0mmol/L 6d	9.00	0.00	–	13.56**	9.00	0.00	–	59.51**
	125mmol/L 6d	4.00-8.00	15.55			1.00-5.00	35.58		
RSPAD	0mmol/L 3d	100.00	0.00	–	10.39**	100.00	0.00	–	15.20**
	125mmol/L 3d	96.05-98.30	1.70			83.49-90.23	6.10		
	0mmol/L 6d	100.00	0.00	–	21.96**	100.00	0.00	–	37.66**
	125mmol/L 6d	84.78-89.37	4.10			60.25-67.71	9.20		
Fm	0mmol/L 3d	3006.00-3801.00	7.80	1.60	3.48**	2984.00-3833.00	10.20	5.84	3.21**
	125mmol/L 3d	2621.30-3134.00	7.20			1058.00-3399.00	37.60		
	0mmol/L 6d	3344.00-3831.00	6.10	1.39	1.67	2843.00-3836.00	11.30	1.34	8.57**
	125mmol/L 6d	2949.00-3675.00	7.70			612.00-1854.00	33.44		
Fv/Fm	0mmol/L 3d	0.79-0.82	1.40	1.55	3.20**	0.74-0.82	3.80	28.53**	1.86*
	125mmol/L 3d	0.76-0.80	1.70			0.37-0.79	24.20		
	0mmol/L 6d	0.81-0.83	0.60	24.87**	2.67*	0.79-0.83	1.80	46.61**	5.45**
	125mmol/L 6d	0.74-0.81	3.30			0.43-0.68	17.30		
Y	0mmol/L 3d	0.03-0.08	32.20	2.02	0.4	0.02-0.06	36.90	1.03	2.03*
	125mmol/L 3d	0.04-0.07	24.20			0.00-0.04	63.80		
	0mmol/L 6d	0.03-0.05	20.10	6.66	-2.73	0.03-0.08	34.60	15.44**	4.98**
	125mmol/L 6d	0.03-0.07	36.90			0.01-0.02	32.10		
NPQ	0mmol/L 3d	1.83-3.98	26.30	3.48	2.45*	1.73-3.65	30.10	1.47	2.47*
	125mmol/L 3d	1.59-2.60	20.10			0.47-2.14	41.00		
	0mmol/L 6d	2.25-3.48	14.90	1.19	-0.59	1.82-3.02	17.30	3.64	6.99**
	125mmol/L 6d	2.18-3.49	15.40			0.67-1.27	20.50		
ETR	0mmol/L 3d	1.48-4.22	32.80	2.11	0.39	1.08-3.18	37.70	1.07	2.00*
	125mmol/L 3d	1.78-3.37	24.00			0.10-1.90	63.90		
	0mmol/L 6d	1.58-2.52	20.20	5.88	-2.62	1.52-4.13	34.90	5.79	4.95**
	125mmol/L 6d	1.20-3.66	35.80			0.09-1.20	61.80		
SNa^+^	0mmol/L 3d	2.22-6.90	35.30	12.34**	-42.55**	2.28-5.49	25.40	120.16**	-23.90**
	125mmol/L 3d	41.20-56.79	10.30			34.99-68.19	19.80		
	0mmol/L 6d	2.03-6.54	30.80	8.92**	-57.37**	2.15-5.39	22.30	303.06**	-25.81**
	125mmol/L 6d	52.23-66.30	6.70			51.19-110.50	18.40		
RNa^+^	0mmol/L 3d	10.67-19.53	21.80	37.88**	-12.52**	12.07-19.98	14.70	171.31**	-9.42**
	125mmol/L 3d	41.74-98.74	29.50			21.03-114.82	41.60		
	0mmol/L 6d	8.67-13.67	9.60	198.20**	-17.65**	9.05-19.43	23.40	40.35**	-16.01**
	125mmol/L 6d	47.14-87.21	23.30			54.06-139.10	27.50		
SK^+^	0mmol/L 3d	21.94-43.47	18.10	1.08	3.14**	22.16-42.92	19.70	1.90	3.58**
	125mmol/L 3d	19.40-37.41	21.10			18.06-36.88	17.50		
	0mmol/L 6d	25.11-43.47	21.30	1.90	5.35**	27.57-44.10	14.90	1.89	8.33**
	125mmol/L 6d	16.15-32.29	22.30			8.98-29.83	35.90		
RK^+^	0mmol/L 3d	12.43-33.46	35.40	2.43	2.95**	12.18-23.26	20.70	1.33	1.57
	125mmol/L 3d	7.98-18.86	32.40			9.26-21.45	26.50		
	0mmol/L 6d	13.49-27.50	22.40	1.77	3.81**	17.73-33.08	19.00	1.25	10.12**
	125mmol/L 6d	9.09-19.44	22.80			5.15-17.19	38.00		
SNa^+^/K^+^	0mmol/L 3d	0.08-0.16	25.40	208.76**	-8.81**	0.09-0.14	16.30	430.00**	-11.63**
	125mmol/L 3d	1.27-2.36	25.80			1.38-2.44	19.80		
	0mmol/L 6d	0.07-0.22	44.00	182.90**	-7.86**	0.08-0.13	14.40	20440.12**	-4.89**
	125mmol/L 6d	1.79-3.56	29.50			2.75-8.46	48.90		
RNa^+^/K^+^	0mmol/L 3d	0.51-1.27	38.20	59.73**	-4.39**	0.66-1.49	34.80	43.61**	-4.30**
	125mmol/L 3d	2.97-10.40	46.60			2.28-9.00	45.20		
	0mmol/L 6d	0.38-0.78	23.90	42.76**	-10.29**	0.34-0.99	39.40	638.66**	-3.31**
	125mmol/L 6d	3.59-6.25	20.50			4.24-20.30	68.70		

**indicates extremely signifificant difference at p < 0.01; * indicates signifificant difference at p <0.05.

### Significance analysis of 13 salt-tolerance indices

**Figure 2:**
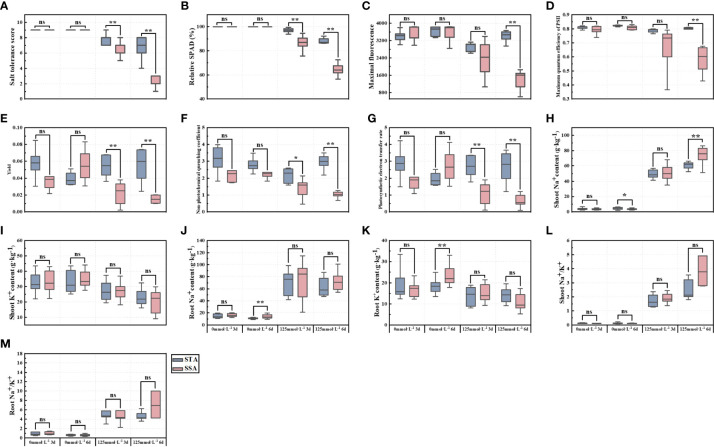
Significance analysis of salt-tolerance indices of *japonica* rice germplasm accessions with different salt tolerances under salt stress. **(A)** STS, **(B)** RSPAD, **(C)** Maximal fluorescence (Fm), **(D)** Maximum quantum efficiency of PSII (Fv/Fm), **(E)** Yield (Y), **(F)** Non-photochemical quenching coefficient (NPQ), **(G)** Photosynthetic electron transfer rate (ETR), **(H)** Shoot Na^+^ content (SNa^+^), **(I)** Shoot K^+^ content (SK^+^), **(J)** Root Na^+^ content (RNa^+^), **(K)** Root K^+^ content (RK^+^), **(L)** Shoot Na^+^/K^+^ (SNa^+^/K^+^), and **(M)** Root Na^+^/K^+^ (RNa^+^/K^+^); **p < 0.01, *p< 0.05; ns, not significant.

Significance analysis revealed that SNa^+^ of STA was significantly higher than that of SSA in the CK group at day 6, whereas RNa^+^ and RK^+^ of SSA were highly significantly higher than those of STA (**Figure 2**). At day 3 after salt stress, STS, RSPAD, Y, and ETR in STA were highly significantly higher than their respective values in SSA, and NPQ was significantly higher in STA than in SSA. STS, RSPAD, Fm, Fv/Fm, Y, NPQ, and ETR were highly significantly higher in STA than in SSA at 6 days after salt stress. Among ionic indices, only SNa^+^ was significantly lower in STA than in SSA, and SK^+^, RK^+^, SNa^+^/K^+^, and RNa^+^/K^+^ were not significantly different between STA and SSA.

### Correlation analysis of 13 salt-tolerance indices

**Figure 3:**
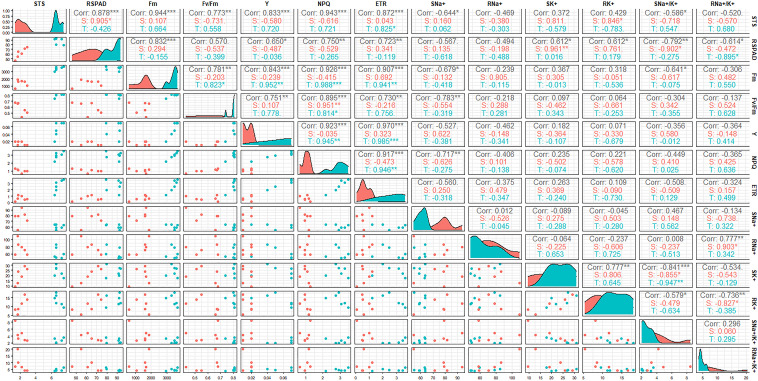
Correlation coefficient matrix between salt-tolerance indices at the seedling stage of *japonica* rice germplasm accessions under salt stress. The correlation coefficient (r^2^) is shown in the figure; T: Salt-tolerant accessions, S: Salt-sensitive accessions, ***p < 0.001, **p < 0.01, *p < 0.05.

Since most of the indices of STA exhibited a highly significant difference compared with SSA at 6 days after salt stress, a correlation analysis of salt tolerance-related indices was conducted using the 12 *japonica* germplasm accessions at day 6 after salt stress (**Figure 3**). STS exhibited highly significant positive correlations with RSPAD, Fm, Fv/Fm, Y, NPQ, and ETR (p < 0.01) and significant negative correlations with SNa^+^ and SNa^+^/K^+^ (p < 0.05). RSPAD exhibited significant or highly significant positive correlations with Fm, Y, NPQ, ETR, SK^+^, and RK^+^ and significant or highly significant negative correlations with SNa^+^/K^+^ and RNa^+^/K^+^. The correlation coefficients between the chlorophyll fluorescence parameters (Fm, Fv/Fm, Y, NPQ, and ETR) were high and all exhibited highly significant positive correlations with each other, indicating a high synergy between these indices. Some chlorophyll fluorescence parameters (Fm, Fv/Fm, and NPQ) exhibited significant or highly significant negative correlations with SNa^+^ and SNa^+^/K^+^. Among ionic parameters, RNa^+^/K^+^ exhibited highly significant positive correlation with RNa^+^ and highly significant negative correlation with RK^+^. SNa^+^/K^+^ exhibited negative correlation of different degrees with both SK^+^ and RK^+^, and SK^+^ exhibited highly significant positive correlation with RK^+^. The correlations of 13 salt tolerance-related indices of STA and SSA were individually analyzed; some indices were observed to be somewhat different between the two germplasm categories. RNa^+^/K^+^ of SSA exhibited a significant positive correlation with RNa^+^ and a significant negative correlation with RK^+^, whereas this correlation was not significant in STA. ETR of STA was significantly or highly significantly positively correlated with STS, Fm, Y, and NPQ; NPQ was positively correlated with Fm, Fv/Fm, and Y; Fv/Fm was positively correlated with Fm. However, these correlations were not significant in SSA.

### PCA of 13 salt-tolerance indices

To investigate the relationship between salt tolerance-related indices and salt-tolerance capacities of rice germplasm accessions, PCA was performed on the 13 indices for 12 *japonica* germplasm accessions (**Table 3**). A total of three principal components (PCs) were extracted with eigenvalues >1.0, explaining 90.254% of the phenotypic variation. PC1 explained 58.471% of the phenotypic variation, and STS, RSPAD, Fm, Y, NPQ, and ETR were the major effectors of this PC with high loadings. In PC1, the loading of STS was the largest (0.972), and all these indices exhibited a positive correlation with salt tolerance, indicating that germplasm accessions with higher PC1 may have stronger salt tolerance. PC2 and PC3 explained 19.509% and 12.273% of the total phenotypic variation, respectively, with SK^+^, RK^+^, and RNa^+^ being the main contributors to these two PCs.

**Table 3:** Eigenvector of load matrix, eigenvalue, and contribution rate of each comprehensive parameter.

Indices	PC1	PC2	PC3
STS	0.972	0.053	-0.075
RSPAD	0.922	-0.260	0.050
Fm	0.943	0.176	0.129
Fv/Fm	0.789	0.450	-0.018
Y	0.873	0.314	-0.206
NPQ	0.940	0.288	-0.092
ETR	0.895	0.270	-0.086
SNa^+^	-0.663	-0.457	-0.360
RNa^+^	-0.454	0.225	0.800
SK^+^	0.496	-0.710	0.395
RK^+^	0.456	-0.796	0.121
SNa^+^/K^+^	-0.684	0.374	-0.555
RNa^+^/K^+^	-0.528	0.663	0.515
Eigenvalue	7.601	2.536	1.596
Proportion of variance (%)	58.471	19.509	12.273
Cumulative proportion (%)	58.471	77.980	90.254

### Comprehensive evaluation of *japonica* rice germplasm accessions in terms of salt tolerance

Using the 3 extracted PCs, the membership function values u (X*_PCj_*) were calculated for all the 12 *japonica* rice germplasm accessions (**Table 4**). Based on their contribution, the weights of the 3 PCs were calculated as 0.648, 0.216, and 0.136, respectively. With membership functions and index weight method, the comprehensive evaluation value *D_CI_* of these germplasm accessions was obtained and ranked. The distribution of *D_CI_* ranged from 0.150 to 0.827, and the *D_CI_* of STA was higher than that of SSA (**Table 4**). The larger the *D_CI_*, the stronger the salt tolerance and vice versa. Depending on the *D_CI_*, Huangluo was determined as a typical salt-tolerant accession with the highest *D_CI_*, and Shanfuliya as a typical salt-sensitive accession with the lowest *D_CI_*.

**Table 4:** Salt-tolerance indices weight, membership function value, *D_CI_* value, and ranking of 12 *japonica* rice germplasm accessions.

Name	u(X*_PC1_*)	u(X*_PC2_*)	u(X*_PC3_*)	*D_CI_*	Ranking
Huangluo	1.000	0.589	0.381	0.827	1
Bertone	0.922	0.877	0.272	0.824	2
Agostono	0.981	0.687	0.290	0.823	3
Cigalon	0.921	0.432	0.715	0.787	4
Banat 2951	0.798	0.395	0.782	0.709	5
Yangbiguangkeludao	0.684	0.386	0.386	0.579	6
Xiannan 22	0.088	1.000	1.000	0.409	7
Koshihikari	0.239	0.495	0.676	0.354	8
Nipponbare	0.278	0.099	0.501	0.270	9
Banat 725	0.281	0.000	0.600	0.263	10
Jianan 8	0.092	0.272	0.375	0.169	11
Shanfuliya	0.000	0.696	0.000	0.150	12
Index weight	0.648	0.216	0.136		

*D_CI_*: Comprehensive evaluation of *D* values of chlorophyll fluorescence and ion content of *japonica* rice germplasm at the seedling stage for salt tolerance.

### Changes in cell membrane permeability and antioxidant enzyme under salt stress

**Figure 4:**
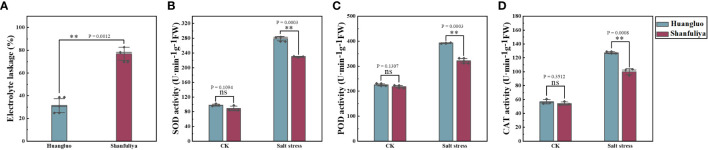
Effect of salt stress on the cell membrane permeability and antioxidant enzyme activity in different salt-tolerant *japonica* rice germplasm. **(A)** Cell membrane permeability at 6 days of salt stress. **(B–D)** SOD activity, POD activity, and CAT activity in the control and salt stress conditions. **p < 0.01, ns, not significant.

The electrolyte leakage rate of salt-tolerant germplasm Huangluo was significantly lower than that of salt-sensitive germplasm Shanfuliya under salt stress (**Figure 4A**). The activities of antioxidant enzymes (SOD, POD, and CAT) exhibited no significant difference between the two germplasms in the CK group (**Figures 4B–D**). Under salt stress, the antioxidant enzyme activity in Huangluo was significantly higher than that in Shanfuliya. Therefore, in Huangluo, the integrity of the cell membrane was probably better maintained.

### Expression analysis of chlorophyll fluorescence and ion-transporter genes in huangluo and shanfuliya

**Figure 5:**
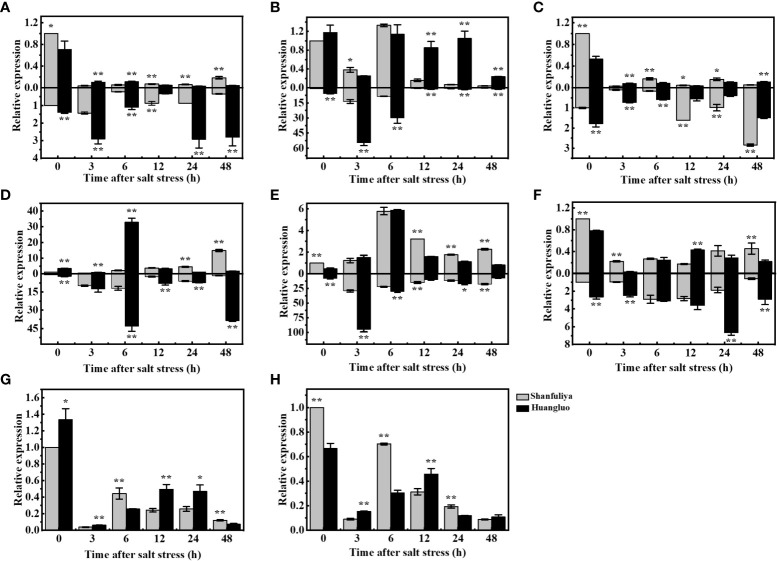
Relative gene expression in *japonica* rice germplasm accessions with different salt tolerances under salt stress at different times. **(A–H)** Relative expression of *OsHKT1;5*, *OsHKT2;1*, *OsAKT2*, *OsHAK21*, *OsNHX1*, *OsSOS1*, *OsHCF222*, and *OsABCI7* under 125 mmol L^−1^ NaCl stress at 0, 3, 6, 12, 24, and 48 h. Relative expression in the rice shoot and root is shown at the top and bottom of the X-axis, respectively. *p < 0.05, **p < 0.01.

The reasons for the differences in salt tolerance between STA and SSA were further assessed using Huangluo and Shanfuliya two germplasm. Their chlorophyll fluorescence and ion-transporter protein gene expression characteristics were analyzed under salt stress. The expressions of chlorophyll fluorescence genes (*OsHCF222* and *OsABCI7*) and ion-transporter protein genes (*OsHKT1;5*, *OsHKT2;1*, *OsAKT2*, *OsHAK21*, *OsNHX1*, and *OsSOS1*) in Huangluo and Shanfuliya exhibited significant or highly significant differences at most time points during 0–48 h of salt stress. The expression of *OsHKT1;5* exhibited an increasing trend at 3 h, gradually decreased at 6 h, and increased again at 24 h in the roots of Huangluo and Shanfuliya (**Figure 5A**). The expression of *OsHKT1;5* was highly significantly higher at 0, 3, 6, 24, and 48 h and lower at 12 h in the roots of Huangluo than in the roots of Shanfuliya. *OsHKT1;5* exhibited a significant decrease after salt stress in the shoots of Huangluo and Shanfuliya. Its expression was significantly higher at 0 h; highly significantly higher at 12, 24, and 48 h; and highly significantly lower at 3 and 6 h in Shanfuliya than in Huangluo. The expression of *OsHKT2;1* differed greatly between Huangluo and Shanfuliya. In the roots, it was extremely significantly higher in Huangluo than in Shanfuliya at all six time points. No significant differences were observed at 0 and 6 h; however, at rest of the time points, the expression was extremely significantly higher in Huangluo than in Shanfuliya in shoots, except at 3 h (**Figure 5B**).

The expression pattern of *OsAKT2* exhibiting a gradual decrease after salt stress followed by a slow increase was clearly different from that of *OsHKT1;5* (**Figure 5C**). The expression of *OsAKT2* was highly significantly higher at 0 and 6 h, significantly higher at 12 and 24 h, and highly significantly lower at 3 and 48 h in the shoots of Shanfuliya than in those of Huangluo. The expression of *OsAKT2* was highly significantly higher at 0, 3, and 6 h and highly significantly lower at 12, 24, and 48 h in the roots of Huangluo than in those of Shanfuliya. *OsHAK21* is a K^+^ transporter protein gene involved in the regulation of Na^+^ and K^+^ homeostasis in rice seedlings. It exhibited differential expression in Huangluo and Shanfuliya. In both shoots and roots of Huangluo, the highest expression of *OsHAK21* was observed at 6 h (**Figure 5D**). The expression of *OsHAK21* was extremely significantly higher in Huangluo roots than in Shanfuliya roots at all time points except 3 h, indicating that *OsHAK21* may play an important role in the response of Huangluo to salt stress. *OsNHX1* responded to salt stress more rapidly, with a rapid increase in its expression at 3 h and a slow decrease after 6 h (**Figure 5E**). *OsSOS1*, the plasma membrane Na^+^/H^+^ exchanger gene, was mainly expressed in the roots. Its expression was significantly higher in Huangluo than in Shanfuliya at 0, 3, 24, and 48 h (**Figure 5F**). The ATP-binding cassette transporter protein OsABCI7 interacts with the protein with high chlorophyll fluorescence OsHCF222 to coregulate cellular ROS stability. Under salt stress, both *OsABCI7* and *OsHCF222* exhibited an initial decreasing trend followed by an increasing trend (**Figures 5G, H**). At 3 and 12 h of salt stress, although the expression of *OsABCI7* and *OsHCF222* was lower than that in the CK group, their expression remained significantly higher in Huangluo than in Shanfuliya.

### Haplotype analysis of chlorophyll fluorescence and ion-transporter genes

Genotypic differentiation is a major cause of differential expression and functional variation of salt tolerance-related genes in rice. Therefore, we selected STA (Huangluo, Bertone) and SSA (Shanfuliya, Nipponbare) for sequencing of chlorophyll fluorescence genes (*OsABCI7* and *OsHCF222*) and ion-transporter protein genes (*OsHKT1;5*, *OsHTK2;1*, *OsHAK21*, *OsAKT2*, *OsNHX1*, and *OsSOS1*). The results revealed that *OsHCF222*, *OsHKT2;1*, and *OsSOS1* did not have polymorphic loci among the four varieties, and the rest five genes did. The sequencing analysis of a set of 29 *japonica* rice accessions (**Supplementary Table 3**) was performed to assess the presence of these polymorphic loci. To clarify the correlation between the polymorphic loci and salt tolerance, the STS values of the 29 germplasm accessions were combined with haplotypes for the significant difference analysis.

**Figure 6:**
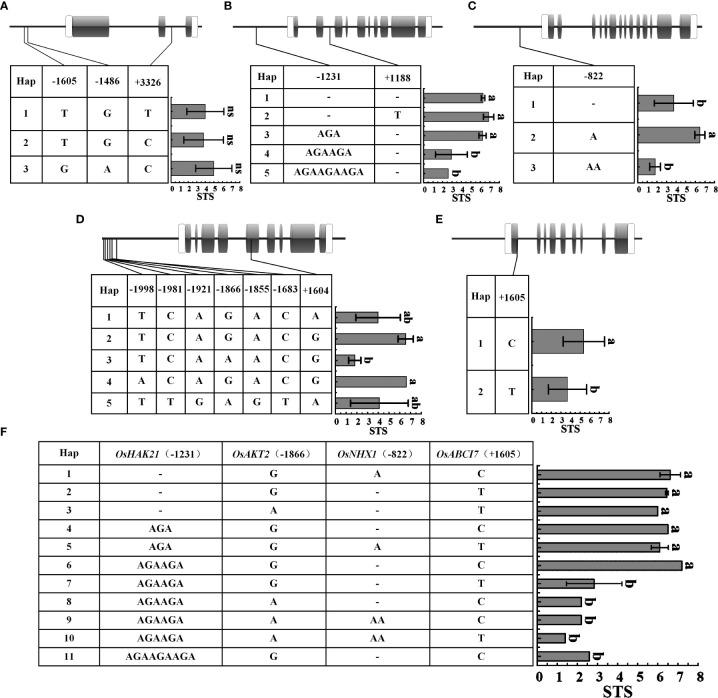
Haplotypes of five genes associated with salt tolerance under salinity stress. **(A–E)** *OsHKT1;5*, *OsHAK21*, *OsNHX1*, *OsAKT2*, and *OsABCI7*. Gray boxes indicate exons; white boxes indicate the 5' and 3′ untranslated region; thick black lines between exons represent introns, and the thick black lines to the left of the 5′ untranslated region represent promoter; **(F)** Multigene combinatorial haplotype analysis of *OsHAK21*, *OsAKT2*, *OsNHX1*, and *OsABCI7*. Different lowercase letters represent significant difference at 5% level (p < 0.05) according to least significant difference (LSD) test.

The differences in STS between different haplotypes of *OsHKT1;5* were not significant (**Figure 6A**). *OsHAK21* had two polymorphic sites in the promoter and intron regions, respectively. Five haplotypes of *OsHAK21* were identified among these 29 accessions, and the STS of Hap1-3 was significantly higher than that of Hap4 and Hap5 (**Figure 6B**). The polymorphic locus located in the promoter of *OsHAK21* (−1231 bp) was an SSR that exhibited four different genotypes and exhibited a significant decrease in salt tolerance with increasing simple sequence repeats (AGA). Hap2, which included some salt-tolerant accessions such as Huangluo and Bertone, was a typical salt-tolerant haplotype, and Hap4 and Hap5 were salt-sensitive haplotypes. *OsNHX1* had only one polymorphic site (−822 bp) in the promoter, and three haplotypes were identified. The STS of Hap2 was significantly higher than that of Hap1 and Hap3 (**Figure 6C**). Hap2 was a typical salt-tolerant haplotype of *OsNHX1*, and either insertion or deletion of this site may significantly affect salt tolerance of rice seedlings. *OsAKT2* had six polymorphic sites in the promoter and one polymorphic site in the exon (**Figure 6D**), and five haplotypes were identified. The results revealed that the STS of Hap2 and Hap4 was significantly higher than that of Hap3. Hap2 and Hap4 were considered to be salt-tolerant haplotypes containing salt-tolerant accessions such as Banat 2951, Bertone, and Huangluo. Among them, Hap2 and Hap3 only had an SNP (G/A) at −1866 bp, which may be a key site for *OsAKT2* to respond to salt stress. *OsABCI7* had only one SNP (T/C) on exon at +1605 bp (**Figure 6E**), and the STS of Hap1 was significantly higher than that of Hap2. Four key loci significantly associated with STS from *OsHAK21*, *OsNHX1*, *OsAKT2*, and *OsABCI7* were extracted for multigene combinatorial haplotype analysis (**Figure 6F**). The results revealed that Hap1–6 were salt-tolerant haplotypes, whose STS was significantly higher than that of salt-sensitive haplotypes (Hap7–11). Hap1 was a typical salt-tolerant haplotype containing five STAs such as Bertone and Huangluo, whereas Hap7 was a typical salt-sensitive haplotype containing 12 SSAs such as Nipponbare, Shanfuliya, and Koshihikari. Hap2 and Hap7 differed only in the SSR of *OsHAK21*, indicating that the SSR located at the *OsHAK21* promoter may have the most significant effect on the STS of the combined haplotypes.

## Discussion

The energy produced by photosynthesis is a key factor supporting crop growth. Photosynthetic pigments, photosystems, electron transport systems, and CO_2_ reduction pathways involved in the photosynthesis process are affected by salt stress (Ashraf and Harris, 2013). Chloroplasts support rice growth by converting light energy into chemical energy, and excess energy is dissipated as heat (NPQ) or emitted as chlorophyll fluorescence (Maxwell and Johnson, 2000). These three processes compete with each other, and under stress, more energy is converted to heat and chlorophyll fluorescence with the reduction in photosynthesis. Assessment of chlorophyll fluorescence parameters can effectively indicate the rate of photochemical reactions and extent of heat dissipation (Tsai et al., 2019). Wang et al. reported that the permeability and function of the thylakoid membrane in chloroplasts were significantly impaired when large amounts of salt ions accumulated in rice cells over a long period (Wang et al., 2009), and the activity of their photosystem and chlorophyll fluorescence were significantly reduced (Faseela et al., 2020). Fm in rice leaves significantly reduced under salt stress compared with the normal treatment (Theerawitaya et al., 2020; Zhu et al., 2022), and Fv/Fm decreased significantly in salt-sensitive varieties (Sarkar et al., 2013; Singh and Sarkar, 2014). In the present study, Fm and Fv/Fm of STA and SSA exhibited significant decreases with increasing duration of salt stress (**Table 2**). Y, ETR, and NPQ of SSA were significantly lower than those on the CK group at 3 days after salt stress and exhibited a highly significant decrease at day 6 of salt stress. Fm, Fv/Fm, Y, ETR, and NPQ of STA at 6 days after salt stress were highly significantly higher than those of SSA (**Figure 2**), indicating that the electron transfer rate and photochemical reaction rate of STA were higher and the PSII complex was less damaged under salt stress. NPQ reflects the level of excess energy converted into heat dissipation. It was reported that NPQ of rice varieties with different salt tolerances increased significantly under salt stress (Moradi and Ismail, 2007), whereas another study reported increased NPQ in Pokkali (salt-tolerant *indica* rice) and decreased NPQ in IR-29 (salt-sensitive *indica* rice) (Lee et al., 2013). In our study, NPQ of SSA was significantly lower than that of the CK group at days 3 and 6 after salt stress. However, NPQ of STA was lower than that of the CK group at day 3, but it significantly improved at day 6 compared with that at day 3 (**Figure 2**). It may be due to lower inhibition of light energy conversion efficiency in STA at the early stage of salt stress, and only limited excess energy was produced for heat dissipation; therefore, NPQ was lower at day 3 after salt stress. With the prolongation of duration of stress, the photosystem conversion efficiency gradually decreased to reduce the damage to the thylakoid membrane from excess energy. In STA, a thermal dissipation mechanism was initiated, which converted the excess energy into thermal energy for dissipation, resulting in an increase in NPQ and a protective effect on the thylakoid membrane.

Na^+^, a mineral element required for rice growth, can be promoted at low Na^+^ concentrations, particularly under K^+^-deficient conditions (Horie et al., 2007). However, high concentrations of Na^+^ can have direct toxic effects on cells, e.g., inhibition of multiple metabolic processes by competing with K^+^, which was required for a variety of biochemical reactions and protein synthesis (Belgaroui et al., 2018). On this basis, Na^+^ produces sustained osmotic and oxidative stresses that damage the integrity of cell membranes and cell walls to inhibit rice growth (Ismail et al., 2014; Voxeur and Höfte, 2016; Zhu, 2016; Yang and Guo, 2018a, YangGuo, 2018b; Zhao et al., 2021). To adapt to saline soil, plants have evolved complex mechanisms to maintain Na^+^ homeostasis, which is mainly controlled by ion-transport proteins (Zhu, 2016; Yang and Guo, 2018a). OsHKT1;5, isolated from the salt-tolerant *indica* rice variety (Nona Bokra), plays an important role in regulating ion homeostasis in *indica* rice (Ren et al., 2005; Hauser and Horie, 2010; Alnayef et al., 2020). In our study, the expression of *OsHKT1;5* was significantly higher in the roots of Huangluo than in those of Shanfuliya (**Figure 5A**), whereas the Na^+^ content was significantly lower in the roots of Huangluo than in those of Shanfuliya at 6 days after salt stress (**Supplementary Figure 1**). This indicated that OsHKT1;5 plays an important role in regulating Na^+^ homeostasis in *japonica* rice. OsHKT2;1, a versatile protein of HKT subgroup II, regulates ion transportation. OsHKT2;1 cotransports Na^+^ and K^+^ under physiological conditions, separately cotransports Na^+^ under high Na^+^ concentration, and takes up Na^+^ to maintain normal rice growth under K^+^-deficient conditions (Jabnoune et al., 2009; Hauser and Horie, 2010; Suzuki et al., 2016; Hartley et al., 2019). However, we observed that the expression of *OsHKT2;1* was significantly higher in Huangluo than in Shanfuliya; additionally, Theerawitaya et al. reported that Pokkali exhibited a significant increase in *OsHKT2;1* expression after salt stress, which was approximately 2.5-fold higher than that under normal growth conditions (Theerawitaya et al., 2020). This may be because of K^+^ deficiency due to high Na^+^ concentrations and the uptake of Na^+^ by OsHKT2;1 to maintain normal growth. Although *OsAKT2* expressed in the shoot phloem loaded and transported K^+^ to the roots for redistribution to alleviate rice salt stress (Tian et al., 2021), our results revealed that the expression of *OsAKT2* was suppressed. This may be a protective mechanism against K^+^ loss in the shoots, causing excessive Na^+^/K^+^ in shoot. In addition, we observed that both *OsNHX1* and *OsHKT2;1* were expressed in the vascular sheaths (Horie et al., 2007; Fukuda et al., 2011), with high synergism (**Figures 5B–E**), speculating that a coexpression network may exist between them; however, so far, no reliable basis is obtained for this inference.

Sequence polymorphism analysis helps us to understand the mechanisms of adaptation to salt stress in different rice germplasms (Oomen et al., 2012). He et al. identified 14 SNPs and 2 indels in the *OsHAK21* promoter and coding region, where four SNPs located in the coding region caused amino acid substitution. Haplotype analysis revealed that 2036T SNP located in the promoter was significantly positively correlated with seed germination under salt stress, and Hap3 containing 2036T SNP was a typical salt-tolerant haplotype (He et al., 2019). In our study, all *japonica* rice germplasm accessions exhibited the 2036C SNP in *OsHAK21* promoter. Interestingly, we observed one SSR sequence in its promoter, and the STS of germplasm accessions gradually decreased with the increase of AGA trinucleotide repeat motifs (**Figure 6B**). The expression of *OsHAK21* was significantly higher in Huangluo (Hap2) than in Shanfuliya (Hap4), indicating that the SSR sequence may affect the salt tolerance of rice seedlings by regulating the expression of *OsHAK21*. We observed that an ABRE cis-acting element located in this SSR region is associated with response to ABA, and *OsHAK21* is involved in the ABA synthesis, ABA signaling pathway-related gene expression, and inhibition of ROS accumulation to regulate salt tolerance in rice (cf. PlantCARE database) (Lescot et al., 2002; He et al., 2019). Similarly, this occurs in the *OsNHX1* promoter. We detected an indel site at *OsNHX1* promoter (−822 bp); Huangluo (Hap2) was A, Shanfuliya (Hap1) was a single-base deletion, and Hap3 was AA. Hap2 had significantly higher STS than the other two haplotypes, and the *OsNHX1* expression of Huangluo was significantly higher than that of Shanfuliya. This indicated that with the insertion or deletion of A, this locus may regulate salt tolerance in rice by affecting the expression of *OsNHX1* (**Figure 6C**). In rice shoots, K^+^ can be loaded into the phloem *via* OsAKT2 and other transporter proteins and transported downward, with approximately 80% returning to rice roots, which helps rice roots maintain Na^+^/K^+^ homeostasis and normal functions (Tian et al., 2021). Haplotype analysis revealed that Hap2 and Hap4 were salt tolerant, and Hap3 was salt sensitive. Only one SNP (−1866 bp) was detected in the promoter between Hap2 and Hap3 (**Figure 6D**), which suggested that the SNP may be a key locus affecting *OsAKT2* expression under salt stress in rice seedlings. Meanwhile, one SNP was detected in *OsAKT2* coding region, resulting in an amino acid substitution (glutamate to glycine, E-G; **Supplementary Figure 2A**); however, this locus did not have significant effect on salt tolerance in rice at the seedling stage. The SNP presented in the exon of *OsABCI7* caused the substitution of valine to alanine (V-A; **Supplementary Figure 2B**), which was not located in the critical domain of OsABCI7 (He et al., 2020). This significantly affected salt tolerance in rice seedlings (**Figure 6E**). Multigene combinatorial haplotype analysis suggested that the polymorphic loci significantly associated with salt tolerance located at *OsHAK21* (−1231 bp), *OsNHX1*(−822 bp), *OsAKT2* (−1866 bp), and *OsABCI7* (+1605 bp) can be used to develop molecular markers for the molecular identification of salt-tolerant rice varieties.

**Figure 7:**
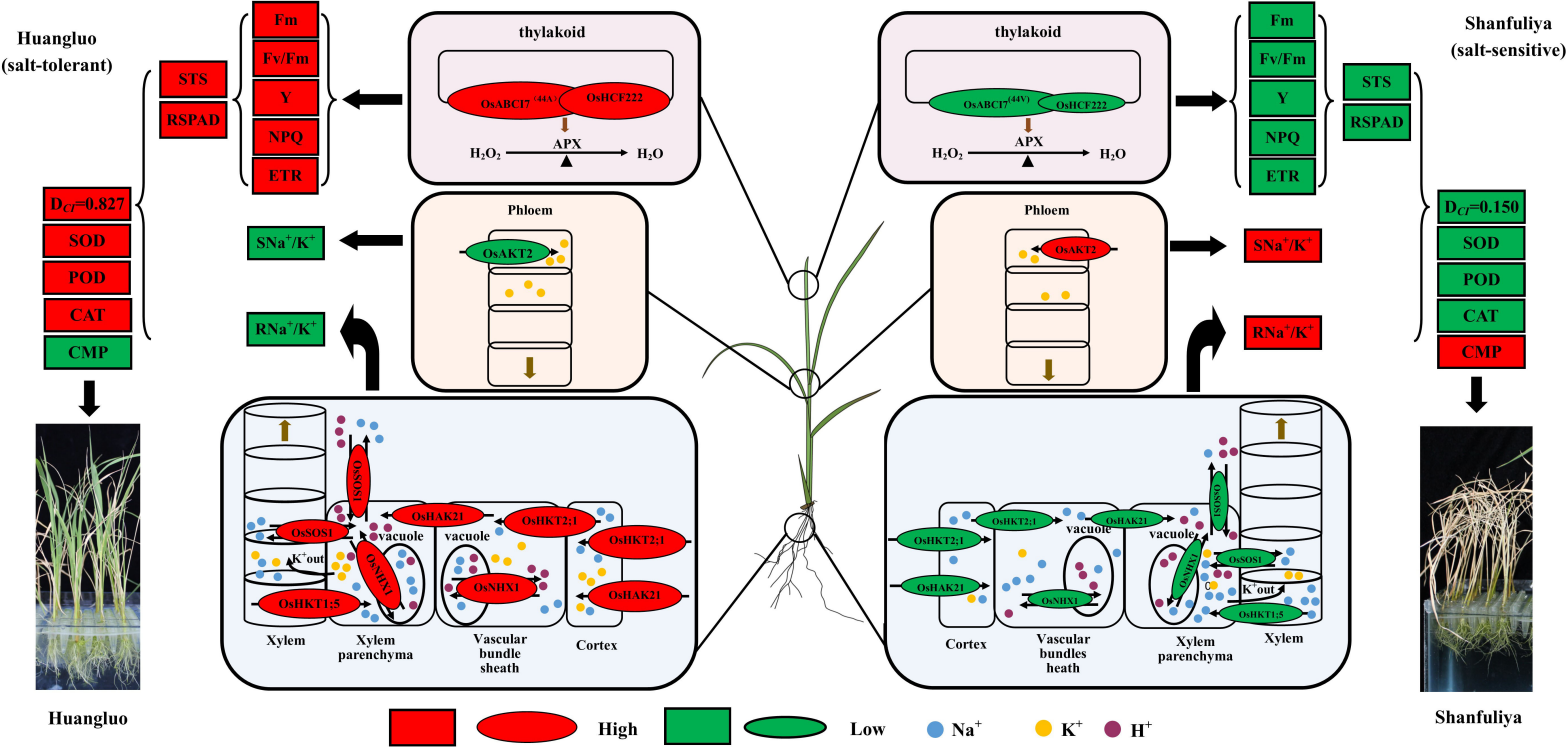
Hypothetical physiological model of *japonica* rice germplasm with different salt tolerances responding to salt stress. The elliptical shapes represent the gene expression levels; red and green colors represent high and low expressions, respectively. The larger the elliptical shapes, the higher the gene expression levels. The boxes represent the measured indices associated with salt tolerance; red and green represent high and low values, respectively.

Considering all the results, we summarized the regulatory patterns of chlorophyll fluorescence and ion-transporter protein genes regulating salt tolerance in different rice germplasm accessions under salt stress (**Figure 7**). When the rice seedlings were subjected to high salt stress, Huangluo (salt-tolerant germplasm) had higher Na^+^ content at day 3 of salt stress than Shanfuliya (salt-sensitive germplasm) (**Supplementary Figure 1**). This may be because *OsHKT2;1* and *OsHAK21* are expressed in the root epidermis and translocated more Na^+^ in Huangluo. With high Na^+^ entering the cells, the expression of *OsNHX1* was rapidly activated on the vesicle membrane within the first 6 h of salt stress in Huangluo (approximately 4 times the expression of *OsNHX1* in Shanfuliya at 3 h) (**Figure 5E**). V-ATP was used up to translocate H^+^ into the cytoplasm, generating an electrochemical potential gradient to translocate Na^+^ into the vesicle, completing ion regionalization to avoid damage to the cell from high Na^+^ content (Wang et al., 2015a, Wang et al., 2019; Goyal et al., 2016). However, OsSOS1, located on the cell membrane, also translocated Na^+^ to the apoplast (Martínez-Atienza et al., 2007; El Mahi et al., 2019). In contrast, the relative expression of *OsNHX1* and *OsSOS1* in Shanfuliya was low, and only limited Na^+^ was regionalized and translocated out of the cell, with high concentrations of Na^+^ retained in the cytoplasm leading to ion toxicity (Apse et al., 1999; Liu et al., 2010). *OsHKT1;5* and *OsSOS1*, which are expressed in the xylem parenchyma, regulated the content of Na^+^ and transported it to shoot through acting antagonistically (El Mahi et al., 2019). The Na^+^ content in the shoot was higher in Huangluo than in Shanfuliya at 3 days of salt stress (**Supplementary Figure 1**). It is possible that the high expression of *OsSOS1* expelled the excess Na^+^ into the xylem. In Huangluo, OsHKT1;5 transported Na^+^ to the xylem parenchyma to counteract some of the function of OsSOS1 and caused membranes depolarization of xylem parenchyma cells, opening K^+^ channels and releasing K^+^ into the xylem vessels (Ren et al., 2005; Hauser and Horie, 2010; El Mahi et al., 2019; Alnayef et al., 2020). Such an antagonistic mechanism not only avoids the transport of large amount of Na^+^ to the shoot but also uses the high concentration of K^+^ in the shoot to neutralize some of the Na^+^ content to reduce the damage to the roots due to high Na^+^ concentration. Therefore, the Na^+^/K^+^ ratio was significantly lower in the shoot of Huangluo than in that of Shanfuliya (**Supplementary Figure 1C**). As the expression of *OsAKT2* was higher in Shanfuliya than in Huangluo (**Figure 5C**), with the shoot K^+^ transported to the roots, the root Na^+^/K^+^ ratio was lower and shoot Na^+^/K^+^ ratio was higher in Shanfuliya than in Huangluo at 3 days of salt stress (**Supplementary Figure 1C**). Concurrently, *OsABCI7* and *OsHCF222* on rice chloroplast thylakoid membrane responded to salt stress with lower expression in Shanfuliya, which could only form a smaller amount of OsABCI7-OsHCF222 protein complex. Therefore, it was unable tocould not scavenge a lot of ROS through the APX pathway, resulting in an excessive accumulation of ROS, damaging the thylakoid membrane, and affecting normal photosynthesis; this was manifested as a decrease in chlorophyll fluorescence parameters (Fm, Fv/Fm, Y, NPQ, and ETR), RSPAD, and STS (He et al., 2020; Zhu et al., 2022). Huangluo could produce more OsABCI7-OsHCF222 protein complex to maintain ROS homeostasis in the chloroplasts with the assistance of the APX pathway.

In summary, Huangluo was better than Shanfuliya in terms of maintenance of intracellular Na^+^ and K^+^ and ROS homeostasis by coordinating multiple ion-transport proteins (El Mahi et al., 2019). Thus, the cells of Huangluo were relatively intact after 6 days of salt stress and could maintain normal function, exhibiting greater salt tolerance. However, the salt tolerance of Shanfuliya was lower, with most of the cells dying after 6 days of salt stress (**Figure 4A**). Therefore, in Shanfuliya, the damage due to salt was more severe, and eventually, the plants died.

## Conclusions

The differential response of salt stress in *japonica* rice germplasm with different salt tolerances mainly resulted from the disparity in chlorophyll fluorescence and ion-transporter protein gene expressions. And some genes’ differential expressions may due to the genotypic variations in promoter regions. We detected four key variations, including an SNP in the coding region of *OsABCI7* and three polymorphic sites in the promoter of *OsHAK21*, *OsNHX1*, and *OsAKT2*, which may play key roles by affecting the response of these genes to salt stress.

## Data availability statement

The original contributions presented in the study are included in the article/**Supplementary Material**, further inquiries can be directed to the corresponding author/s.

## Author contributions

LT supervised and designed the experiments. JS guided the research and wrote the manuscript. JS, HY, CQ, CZ, TB, HD, SM, NW, CL, YZ, TM and PL performed the experiments and data analysis. All authors have read and approved the final manuscript. All authors contributed to the article and approved the submitted version.

## References

[B1] Alnayef M. Solis C. Shabala L. Ogura T. Chen Z. Bose J.. (2020). Changes in expression level of *OsHKT1;5* alters activity of membrane transporters involved in K^+^ and Ca^2+^ acquisition and homeostasis in salinized rice roots. Int. J. Mol. Sci. 21, 4882. doi: 10.3390/ijms21144882 32664377 PMC7402344

[B2] Apse M. P. Aharon G. S. Snedden W. A. Blumwald E. (1999). Salt tolerance conferred by overexpression of a vacuolar Na^+^/H^+^ antiport in *Arabidopsis* . Science 285, 1256–1258. doi: 10.1126/science.285.5431.1256 10455050

[B3] Ashraf M. Harris P. J. C. (2013). Photosynthesis under stressful environments: An overview. Photosynthetica 51, 163–190. doi: 10.1007/s11099-013-0021-6

[B4] Bassil E. Zhang S. Gong H. Tajima H. Blumwald E. (2018). Cation specificity of vacuolar NHX-type cation/H^+^ antiporters. Plant Physiol. 179, 616–629. doi: 10.1104/pp.18.01103 30498025 PMC6426403

[B5] Belgaroui N. Lacombe B. Rouached H. Hanin M. (2018). Phytase overexpression in *Arabidopsis* improves plant growth under osmotic stress and in combination with phosphate deficiency. Sci. Rep. 8, 1137. doi: 10.1038/s41598-018-19493-w 29348608 PMC5773496

[B6] El Mahi H. Pérez-Hormaeche J. De Luca A. Villalta I. Espartero J. Gámez-Arjona F.. (2019). A critical role of sodium flux *via* the plasma membrane Na^+^/H^+^ exchanger SOS1 in the salt tolerance of rice. Plant Physiol. 180, 1046–1065. doi: 10.1104/pp.19.00324 30992336 PMC6548274

[B7] Faseela P. Sinisha A. K. Brestic M. Puthur J. T. (2020). Chlorophyll a fluorescence parameters as indicators of a particular abiotic stress in rice. Photosynthetica 58, 293–300. doi: 10.32615/ps.2019.147

[B8] Feng H. Tang Q. Cai J. Xu B. Xu G. Yu L. (2019). Rice OsHAK16 functions in potassium uptake and translocation in shoot, maintaining potassium homeostasis and salt tolerance. Planta 250, 549–561. doi: 10.1007/s00425-019-03194-3 31119363

[B9] Fukuda A. Nakamura A. Hara N. Toki S. Tanaka Y. (2011). Molecular and functional analyses of rice NHX-type Na^+^/H^+^ antiporter genes. Planta 233, 175–188. doi: 10.1007/s00425-010-1289-4 20963607

[B10] Fukuda A. Nakamura A. Tagiri A. Tanaka H. Miyao A. Hirochika H.. (2004). Function, intracellular localization and the importance in salt tolerance of a vacuolar Na^+^/H^+^ antiporter from rice. Plant Cell Physiol. 45, 146–159. doi: 10.1093/pcp/pch014 14988485

[B11] Ganie S. A. Molla K. A. Henry R. J. Bhat K. V. Mondal T. K. (2019). Advances in understanding salt tolerance in rice. Theor. Appl. Genet. 132, 851–870. doi: 10.1007/s00122-019-03301-8 30759266

[B12] Goyal E. Amit S. K. Singh R. S. Mahato A. K. Chand S. Kanika K. (2016). Transcriptome profiling of the salt-stress response in *Triticum aestivum* cv. kharchia local. Sci. Rep. 6, 27752. doi: 10.1038/srep27752 27293111 PMC4904219

[B13] Hartley T. N. Thomas A. S. Maathuis F. J. M. (2019). A role for the OsHKT2;1 sodium transporter in potassium use efficiency in rice. J. Exp. Bot. 71, 699–706. doi: 10.1093/jxb/erz113 PMC6946003 30854552

[B14] Hassani A. Azapagic A. Shokri N. (2021). Global predictions of primary soil salinization under changing climate in the 21st century. Nat. Commun. 12, 6663. doi: 10.1038/s41467-021-26907-3 34795219 PMC8602669

[B15] Hauser F. Horie T. (2010). A conserved primary salt tolerance mechanism mediated by HKT transporters: A mechanism for sodium exclusion and maintenance of high K^+^/Na^+^ ratio in leaves during salinity stress. Plant Cell Environ. 33, 552–565. doi: 10.1111/j.1365-3040.2009.02056.x 19895406

[B16] He Y. Shi Y. Zhang X. Xu X. Wang H. Li L.. (2020). The OsABCI7 transporter interacts with OsHCF222 to stabilize the thylakoid membrane in rice. Plant Physiol. 184, 283–299. doi: 10.1104/pp.20.00445 32661060 PMC7479889

[B17] He Y. Yang B. He Y. Zhan C. Cheng Y. Zhang J.. (2019). A quantitative trait locus, *qSE*3, promotes seed germination and seedling establishment under salinity stress in rice. Plant J. 97, 1089–1104. doi: 10.1111/tpj.14181 30537381 PMC6850641

[B18] Horie T. Costa A. Kim T. H. Han M. J. Horie R. Leung H. Y.. (2007). Rice OsHKT2;1 transporter mediates large Na^+^ influx component into K^+^-starved roots for growth. EMBO J. 26, 3003–3014. doi: 10.1038/sj.emboj.7601732 17541409 PMC1894770

[B19] Ismail A. Takeda S. Nick P. (2014). Life and death under salt stress: same players, different timing? J. Exp. Bot. 65, 2963–2979. doi: 10.1093/jxb/eru159 24755280

[B20] Jabnoune M. Espeout S. Mieulet D. Fizames C. Verdeil J. L. Conéjéro G.. (2009). Diversity in expression patterns and functional properties in the rice HKT transporter family. Plant Physiol. 150, 1955–1971. doi: 10.1104/pp.109.138008 19482918 PMC2719131

[B21] Lee M. H. Cho E. J. Wi S. G. Bae H. Kim J. E. Cho J. Y.. (2013). Divergences in morphological changes and antioxidant responses in salt-tolerant and salt-sensitive rice seedlings after salt stress. Plant Physiol. Biochem. 70, 325–335. doi: 10.1016/j.plaphy.2013.05.047 23811121

[B22] Lescot M. Déhais P. Thijs G. Marchal K. Moreau Y. Peer Y. V. D.. (2002). PlantCARE, a database of plant cis-acting regulatory elements and a portal to tools for in silico analysis of promoter sequences. Nucleic Acids Res. 30, 325–327. doi: 10.1093/nar/30.1.325 11752327 PMC99092

[B23] Li H. Li Y. Si Y. Du C. Zhou X. Liu M.. (2020). Principal component analysis and comprehensive evaluation of saline-alkaline tolerance related traits of northern *japonica* rice. J. Nucl. Agric. Sci. 34, 1862–1871. doi: 10.11869/j.issn.100-8551.2020.08.1862

[B24] Liu C. Mao B. Yuan D. Chu C. Duan M. (2022). Salt tolerance in rice: Physiological responses and molecular mechanisms. Crop J. 10, 13–25. doi: 10.1016/j.cj.2021.02.010

[B25] Liu S. Zheng L. Xue Y. Zhang Q. Wang L. Shou H. (2010). Overexpression of *OsVP1* and *OsNHX1* increases tolerance to drought and salinity in rice. J. Plant Biol. 53, 444–452. doi: 10.1007/s12374-010-9135-6

[B26] Lu X. Min W. Shi Y. Tian L. Li P. Ma T.. (2022). Exogenous melatonin alleviates alkaline stress by removing reactive oxygen species and promoting antioxidant defence in rice seedlings. Front. Plant Sci. 13, 849553 . doi: 10.3389/fpls.2022.849553 35356121 PMC8959771

[B27] Lutts S. Kinet J. M. Bouharmont J. (1996). NaCl-induced senescence in leaves of rice (*Oryza sativa* L.) cultivars differing in salinity resistance. Ann. Bot. 78, 389–398. doi: 10.1006/anbo.1996.0134

[B28] Ma Y. C. Augé R. M. Dong C. Cheng Z. M. (2016). Increased salt tolerance with overexpression of cation/proton antiporter 1 genes: a meta-analysis. Plant Biotechnol. J. 15, 162–173. doi: 10.1111/pbi.12599 27383431 PMC5258863

[B29] Martínez-Atienza J. Jiang X. Garciadeblas B. Mendoza I. Zhu J. K. Pardo J. M.. (2007). Conservation of the salt overly sensitive pathway in rice. Plant Physiol. 143, 1001–1012. doi: 10.1104/pp.106.092635 17142477 PMC1803719

[B30] Ma S. Tian R. Hu H. Lv J. Tian L. Luo C.. (2020). Comprehensive evaluation and selection of rice (*Oryza sativa japonica*) germplasm for saline tolerance at seedling stage. J. Plant Genet. Resour 21, 1089–1101. doi: 10.13430/j.cnki.jpgr.20200115001

[B31] Maxwell K. Johnson G. N. (2000). Chlorophyll fluorescence–a practical guide. J. Exp. Bot. 51, 659–668. doi: 10.1093/jxb/51.345.659 10938857

[B32] Moradi F. Ismail A. M. (2007). Responses of photosynthesis, chlorophyll fluorescence and ROS-scavenging systems to salt stress during seedling and reproductive stages in rice. Ann. Bot. 99, 1161–1173. doi: 10.1093/aob/mcm052 17428832 PMC3243573

[B33] Oomen R. J. F. J. Benito B. Sentenac H. Rodríguez-Navarro A. Talón M. Véry A. A.. (2012). HKT2;2/1, a K^+^-permeable transporter identified in a salt-tolerant rice cultivar through surveys of natural genetic polymorphism. Plant J. 71, 750–762. doi: 10.1111/j.1365-313x.2012.05031.x 22530609

[B34] Ren Z. H. Gao J. P. Li L. G. Cai X. L. Huang W. Chao D. Y.. (2005). A rice quantitative trait locus for salt tolerance encodes a sodium transporter. Nat. Genet. 37, 1141–1146. doi: 10.1038/ng1643 16155566

[B35] Sarkar R. K. Mahata K. R. Singh D. P. (2013). Differential responses of antioxidant system and photosynthetic characteristics in four rice cultivars differing in sensitivity to sodium chloride stress. Acta Physiol. Plant 35, 2915–2926. doi: 10.1007/s11738-013-1322-x

[B36] Shen Y. Shen L. Shen Z. Jing W. Ge H. Zhao J.. (2015). The potassium transporter OsHAK21 functions in the maintenance of ion homeostasis and tolerance to salt stress in rice. Plant Cell Environ. 38, 2766–2779. doi: 10.1111/pce.12586 26046379

[B37] Singh D. P. Sarkar R. K. (2014). Distinction and characterisation of salinity tolerant and sensitive rice cultivars as probed by the chlorophyll fluorescence characteristics and growth parameters. Funct. Plant Biol. 41, 727–736. doi: 10.1071/FP13229 32481027

[B38] Suzuki K. Costa A. Nakayama H. Katsuhara M. Shinmyo A. Horie T. (2016). OsHKT2;2/1-mediated Na^+^ influx over K^+^ uptake in roots potentially increases toxic Na^+^ accumulation in a salt-tolerant landrace of rice Nona Bokra upon salinity stress. J. Plant Res. 129, 67–77. doi: 10.1007/s10265-015-0764-1 26578190

[B39] Theerawitaya C. Tisarum R. Samphumphuang T. Takabe T. Cha-um S. (2020). Expression levels of the Na^+^/K^+^ transporter OsHKT2;1 and vacuolar Na^+^/H^+^ exchanger OsNHX1, Na enrichment, maintaining the photosynthetic abilities and growth performances of indica rice seedlings under salt stress. Physiol. Mol. Biol. Plants 26, 513–523. doi: 10.1007/s12298-020-00769-3 32205927 PMC7078393

[B40] Tian Q. Shen L. Luan J. Zhou Z. Guo D. Shen Y.. (2021). Rice shaker potassium channel OsAKT2 positively regulates salt tolerance and grain yield by mediating K^+^ redistribution. Plant Cell Environ. 44, 2951–2965. doi: 10.1111/pce.14101 34008219

[B41] Tian L. Tan L. Liu F. Cai H. Sun C. (2011). Identification of quantitative trait loci associated with salt tolerance at seedling stage from *Oryza rufipogon* . J. Genet. Genomics 38, 593–601. doi: 10.1016/j.jgg.2011.11.005 22196402

[B42] Tian L. Wang B. Zhang X. Wang N. Pu Z. Dong Y.. (2014). Effects of saline-alkail soil improved by desulfurized gypsum on seedling quality, root features and membrane permeability of rice. Guangdong Agric. Sci. 41, 1–6. doi: 10.16768/j.issn.1004-874x.2014.21.014

[B43] Tsai Y. C. Chen K. C. Cheng T. S. Lee C. Lin S. H. Tung C. W. (2019). Chlorophyll fluorescence analysis in diverse rice varieties reveals the positive correlation between the seedlings salt tolerance and photosynthetic efficiency. BMC Plant Biol. 19, 403. doi: 10.1186/s12870-019-1983-8 31519149 PMC6743182

[B44] Voxeur A. Höfte H. (2016). Cell wall integrity signaling in plants: “To grow or not to grow that’s the question”. Glycobiology 26, 950–960. doi: 10.1093/glycob/cww029 26945038

[B45] Wang N. Chen Y. Tian L. Zhang D. Wang R. Yang M.. (2015). Correlation between root morphological characteristics of *japonica* rice germplasm and salt tolerance at seedling stage. Guangdong Agric. Sci. 42, 1–10. doi: 10.16768/j.issn.1004-874x.2015.10.021

[B46] Wang R. L. Hua C. Zhou F. Zhou Q. C. (2009). Effects of NaCl stress on photochemical activity and thylakoid membrane polypeptide composition of a salt-tolerant and a salt-sensitive rice cultivar. Photosynthetica 47, 125–127. doi: 10.1007/s11099-009-0019-2

[B47] Wang J. Li B. Meng Y. Ma X. Lai Y. Si E.. (2015a). Transcriptomic profiling of the salt-stress response in the halophyte *Halogeton glomeratus* . BMC Genom. 16, 169. doi: 10.1186/s12864-015-1373-z PMC4363069 25880042

[B48] Wang J. Qiu N. Wang P. Zhang W. Yang X. Chen M.. (2019). Na^+^ compartmentation strategy of Chinese cabbage in response to salt stress. Plant Physiol. Biochem. 140, 151–157. doi: 10.1016/j.plaphy.2019.05.001 31103797

[B49] Wei H. Wang X. He Y. Xu H. Wang L. (2021). Clock component OsPRR73 positively regulates rice salt tolerance by modulating OsHKT2;1-mediated sodium homeostasis. EMBO J. 40, e105086. doi: 10.15252/embj.2020105086 33347628 PMC7849171

[B50] Wu H. Zhang X. Giraldo J. P. Shabala S. (2018). It is not all about sodium: Revealing tissue specificity and signalling roles of potassium in plant responses to salt stress. Plant Soil 431, 1–17. doi: 10.1007/s11104-018-3770-y

[B51] Yang Y. Guo Y. (2018a). Elucidating the molecular mechanisms mediating plant salt-stress responses. New Phytol. 217, 523–539. doi: 10.1111/nph.14920 29205383

[B52] Yang Y. Guo Y. (2018b). Unraveling salt stress signaling in plants. J. Integr. Plant Biol. 60, 796–804. doi: 10.1111/jipb.12689 29905393

[B53] Zhao C. Jiang W. Zayed O. Liu X. Tang K. Nie W.. (2021). The LRXs-RALFs-FER module controls plant growth and salt stress responses by modulating multiple plant hormones. Natl. Sci. Rev. 8, nwaa149. doi: 10.1093/nsr/nwaa149 34691553 PMC8288382

[B54] Zhu J. K. (2016). Abiotic stress signaling and responses in plants. Cell 167, 313–324. doi: 10.1016/j.cell.2016.08.029 27716505 PMC5104190

[B55] Zhu C. Song J. Bai T. Wang N. Ma S. Pu Z.. (2022). Effects of NaCl stress on the chlorophyll fluorescence characteristics of seedlings of *japonica* rice germplasm with different salt tolerances. Sci. Agric. Sin. 55, 2509–2525. doi: 10.3864/j.issn.0578-1752.2022.13.003

