# Supplementary material for: Natural variations of chlorophyll fluorescence and ion transporter genes influenced the differential response of japonica rice germplasm with different salt tolerances
Source: Front Plant Sci. 2023 Mar 17;14:1095929. doi: 10.3389/fpls.2023.1095929 (PMC10063860; doi:10.3389/fpls.2023.1095929)
Supplement: Supplementary file 1 [file DataSheet_1.docx]

Supplementary Material

# Supplementary Figures and Tables

## Supplementary Tables

| **Gene** | **RGAP ID** | **Forward primer** | **Reverse primer** | **Product Size** |
| --- | --- | --- | --- | --- |
| *OsHKT1;5* | [*LOC_Os01g20160*](http://rice.uga.edu/cgi-bin/ORF_infopage.cgi?orf=LOC_Os01g20160) | TGGTCAGGCATGGAAGATAGAA | GAAATGCCCCATTAATTAGTAACG | 86 |
| *OsHKT2;1* | [*LOC_Os06g48810*](http://rice.uga.edu/cgi-bin/ORF_infopage.cgi?orf=LOC_Os06g48810) | CTGCTGGTTCTCTGGTACATAAG | GTGACCGAGAATGAGAAGAGTG | 95 |
| *OsAKT2* | [*LOC_Os05g35410*](http://rice.uga.edu/cgi-bin/ORF_infopage.cgi?orf=LOC_Os05g35410) | GCTATGGCGTCTCAGGAAGG | CGCGATCAGGTAGTACAGGC | 148 |
| *OsHAK21* | [*LOC_Os03g37930*](http://rice.uga.edu/cgi-bin/ORF_infopage.cgi?orf=LOC_Os03g37930) | GCGTAGCCCTGTTCTACACTGA | CGTTGGGATCTTCTCGATGAG | 77 |
| *OsNHX1* | [*LOC_Os07g47100*](http://rice.uga.edu/cgi-bin/ORF_infopage.cgi?orf=LOC_Os07g47100) | TCCGGATGCTCCTCACCA | GGGGAGAAGGGCACGAAC | 112 |
| *OsSOS1* | *LOC_Os12g44360* | CTCCGTGCTCATAGAATCGC | ATACTCACTCAAGTGGGTCAATACC | 207 |
| *OsHCF222* | [*LOC_Os03g30092*](http://rice.uga.edu/cgi-bin/ORF_infopage.cgi?orf=LOC_Os03g30092) | TAGAAGGTGGTTGGAGGGG | GATCACCGCACTTCTTGCA | 172 |
| *OsABCI7* | [*LOC_Os11g29850*](http://rice.uga.edu/cgi-bin/ORF_infopage.cgi?orf=LOC_Os11g29850) | ACCGCTGGAACGAAGAAACA | TGTCCCGGAGGAATGGCTTA | 119 |
| *OsActin* | [*LOC_Os03g50885*](http://rice.uga.edu/cgi-bin/ORF_infopage.cgi?orf=LOC_Os03g50885) | GACCTTCAACACCCCTGCTA | ACAGTGTGGCTGACACCATC | 114 |

**Table S1:** qRT-PCR primers used for gene expression analysis

**Table S2:** PCR primers used for gene sequencing

| **Gene** | **Forward primer** | **Reverse primer** | **Purpose** |
| --- | --- | --- | --- |
| *OsHCF222.1* | CATCAATGGCGGAAGACA | GGGTTGGGACTGGGAGT | Full-length genes sequencing for Huangluo, Bertone, Shanfuliya and Nipponbare |
| *OsHCF222.2* | CAAATGGCACGAAAGCG | TTCTGGAGGAAGCGGAGA |  |
| *OsHCF222.3* | TCCCAGTCCCAACCCTG | CGAACGGATAATGAGTAGTAGTGT |  |
| *OsHCF222.4* | TGACCTGCGTCATCCC | CAGTCTAGCCTACCAACAAA |  |
| *OsHCF222.5* | ACCCAAGCATTCTCATT | ACAGTAATCTGGCTCAA |  |
| *OsHCF222.6* | AAACGCATAGACCCTT | TGCTGGCATCATACAG |  |
| *OsHKT2;1.1* | TAGATCCCACCCTACAA | TACCCGAGAACAAAGC |  |
| *OsHKT2;1.2* | GGCGTCCAAGCTATCC | ACCTGCTCAGCGTCAA |  |
| *OsHKT2;1.3* | TAGGCTCTAATGCTCTTG | TGCCGAGTTGATTGTAT |  |
| *OsHKT2;1.4* | TGACGAGCATTTACCAT | TTCGCAAACGAGGAGA |  |
| *OsHKT2;1.5* | ACCATAAGCACAACCCAG | CCAAGTTCTGCACCACTA |  |
| *OsHKT2;1.6* | TGAGGAGCTACAGTATGAT | ACTAGCAGCAACTTTCC |  |
| *OsHKT2;1.7* | ATGGGAATGTAGGGC | GACTGAAACTGGAGGTAA |  |
| *OsHKT2;1.8* | CTGAAGCCTGAACAAA | TCTCCGAGTAATATGAGAA |  |
| *OsHKT1;5.1* | TTGTTAGAGGCACATTAC | GGAGCCGACACTTGAT |  |
| *OsHKT1;5.2* | CTGAAACGGATGGAGC | GGCATATCCCAACTCAA |  |
| *OsHKT1;5.3* | TCTTGTCATCGCCTATC | GCAACAGCAGGAGGTAT |  |
| *OsHKT1;5.4* | GATTTACGCACATGAGAC | ACTAGGGTAGACGAAGGT |  |
| *OsHKT1;5.5* | TGGCTTCCTAATTCCTATAAAC | TGAACGACACCATCCCT |  |
| *OsHKT1;5.6* | GAGGTAGAGCTAGGGTTAGG | TGTCCCAGGCCAGAGTA |  |
| *OsHKT1;5.7* | TTATACTGCGTGAACCT | TCCGTGGATGTGGATTT |  |
| *OsHKT1;5.8* | CATTTCTCCTCGTTTT | ACTAGCATGTCCTTTGT |  |
| *OsHKT1;5.9* | AATAACGCCTAACTGC | TCTCAACCATTCACCC |  |
| *OsAKT2.1* | TGACCCACTGGACCCT | TCACCCTCACTGACCTGTAT |  |
| *OsAKT2.2* | CTTTGGCTCCATACCG | GTGAGACGTGCGTGAAT |  |
| *OsAKT2.3* | CAGGCGTGTCATCTCG | CCAAAGGAAACAGAACCC |  |
| *OsAKT2.4* | AGTAGGTGCTGAGAAGTCG | TGAGGTAGGCGGTGAG |  |
| *OsAKT2.5* | TGGCGTCTCAGGAAGG | GCATGTTTACTGTAGCACC |  |
| *OsAKT2.6* | ACCGCCTACCTCATCG | TCCTCGGGCTTGCTCT |  |
| *OsAKT2.7* | AATTACTTTGCGAGACGAA | GTACTAGCCTTACCAGTGCA |  |
| *OsAKT2.8* | GAGCAAGCCCGAGGACA | GGAGAACCAGCACGCAGT |  |
| *OsAKT2.9* | GACTGCGTGCTGGTTCTC | CGCTCCTTCGTCGTTCA |  |
| *OsAKT2.10* | AGCCACAACGAGCAGC | ACTCAAATCCCTCCAAGC |  |
| *OsAKT2.11* | CCGATGATTTCTGCTC | GAACGTGGTGCTTCC |  |
| *OsHAK21.1* | GGTGGAGGTCGTCGTCT | TGGCTTCATCGGTTCA |  |
| *OsHAK21.2* | AAGAGGGTTTCACTTCTAC | CTATGCGTGCGGTTTG |  |
| *OsHAK21.3* | CTCGCCAAAGTAAACG | GGAACGCCAGACTCAT |  |
| *OsHAK21.4* | CGAATAGCGATGGTAA | ATCTTCCGCCTGATGG |  |
| *OsHAK21.5* | CATAGAAAACGACGAATAA | GGATGGTGAGCAGGAA |  |
| *OsHAK21.6* | GTGAGCCTGATCCCTAA | TGAAAGTTCCCAGCAA |  |
| *OsHAK21.7* | CCGTCTAGTACAACGAA | GAGTAATACCGATGGAA |  |
| *OsHAK21.8* | GAAGGAAGGATGGGTC | TAGACAGCCTCCGTTG |  |
| *OsHAK21.9* | TTGCGTTGTGACTGTTAG | AAGCGATGAGTGAGGC |  |
| *OsHAK21.10* | AGCGACCGTCTACCAC | CAGCCAAGACTTGTGC |  |
| *OsNHX1.1* | TTTCTGCTTCCTGCTAG | AATGGTGATAGGGATTTAG |  |
| *OsNHX1.2* | CAACGATGTAGGATGAGA | TTTTGGTTTGTGGAAA |  |
| *OsNHX1.3* | ACGCCAGAATTGAAAGG | GATTGAAACGAGGAACG |  |
| *OsNHX1.4* | CCACGTTCCTCGTTTCA | CAAGTCAAGGTATGCCAGT |  |
| *OsNHX1.5* | ATCTACCTCCTCCCTCC | AAGGTTACACGGAACAAC |  |
| *OsNHX1.6* | TAGATTTGAAGTAGGGTTG | ATGAGGGTTTGTTTGG |  |
| *OsNHX1.7* | TTCGGTGAAGGTGTTG | GCTGTCTCCAGGTTATT |  |
| *OsNHX1.8* | TTTCCCATTCGCAGAC | TAGCTCGTAAACAATCAAGT |  |
| *OsNHX1.9* | TTTTGTCCCTACTATCTTG | GCAAACCATGCCATAC |  |
| *OsNHX1.10* | TGGAGGAAGATGAACAG | ACTAAATAGGGCCTAACA |  |
| *OsSOS1.1* | ATAGATAGAGGTGGGC | AACAGGGAACTTGGA |  |
| *OsSOS1.2* | CTACTAGCGGCTCAAA | CACTTCAAACGCAGAA |  |
| *OsSOS1.3* | GAAGGCGGTTCTGC | ACACCAGGTCCAGCA |  |
| *OsSOS1.4* | TGGCACAAATGGTGTT | ATTCCACATACGCTCC |  |
| *OsSOS1.5* | TGCCTGCTGCTCAC | TATGGGAAGAAGACAAA |  |
| *OsSOS1.6* | TGGGTGAAACTTTGG | GATAATCCCTTCTACTGG |  |
| *OsSOS1.7* | AGTAGCCAGTCATTTCAC | CATCAAGATAGGACCACA |  |
| *OsSOS1.8* | TAGGGCTAGAGCAAAG | CAATAGTCGTGGAAGG |  |
| *OsSOS1.9* | CCTTCCACGACTATTGA | TCGCCACTGGCTAC |  |
| *OsSOS1.10* | ACTGGTGGCATCGTG | CAATTATGACTTAGTGGGT |  |
| *OsSOS1.11* | CCTGCTCCAAAGTCC | TCTTATTGAAAGCCAAG |  |
| *OsSOS1.12* | AACTTGGTCCTCCTGC | GCCGTCTTGCGATT |  |
| *OsSOS1.13* | AGAAGGACGAATAACTCA | TCCCAGCACAACAACT |  |
| *OsSOS1.14* | TGGAAGCCTGGTTG | ACTCAAGTGGGTCAATAC |  |
| *OsSOS1.15* | AACTGCAATGGGACTA | CCAATCAGCACCTCA |  |
| *OsSOS1.16* | CTGCGATGCGTGAT | CCCTTTCCTCCTTGTA |  |
| *OsSOS1.17* | GATAAAGTTACAAGGAGGAA | CGGCATTTGGTGGT |  |
| *OsSOS1.18* | CTACATTAAGGGCGAAGA | CTGACGGAAGGTGAGC |  |
| *OsSOS1.19* | CCCTTGAACTGCCTCG | TACGCTAATGACTTTCTTGT |  |
| *OsABCI7.1* | TCAACTGCTCCACGAAA | GCCTTGTTACCCTCCC |  |
| *OsABCI7.2* | CATTAAGGCTAGACCTTTGCG | GGTTCCTCCACTTTCTCACTCT |  |
| *OsABCI7.3* | TCAGGCGATGAGGTGGC | TGAGGTTTGGGTCGGGTC |  |
| *OsABCI7.4* | GAAGAGTGAGAAAGTGGAGGAA | TACCCAACTGCTGAGACTGAAA |  |
| *OsABCI7.5* | CGTGGATTCAGGGAGG | AGATGGAGGATAAGAGGGA |  |
| *OsABCI7.6* | CGCCGTTCTACGAGTC | TCATTAGTCATTACCGAGT |  |
| *OsABCI7.7* | GTCTATCGGAACCTACCC | ATGATACGATGCCCAC |  |
| *OsABCI7.8* | TCTTTGTGGGCATCG | TGGAATAGTCAGGCAGTA |  |
| *OsABCI7.9* | CAAATCAAGGATGGTCGTA | TTAGCAATGCCAAGAGT |  |
| *OsHKT1;5.a* | TTGTTAGAGGCACATTAC | GGAGCCGACACTTGAT | Polymorphic sites sequencing for 29 *japonica* rice germplasm |
| *OsHKT1;5.b* | TTATACTGCGTGAACCT | TCCGTGGATGTGGATTT |  |
| *OsAKT2.a* | TGACCCACTGGACCCT | TCACCCTCACTGACCTGTAT |  |
| *OsAKT2.b* | AATTACTTTGCGAGACGAA | GTACTAGCCTTACCAGTGCA |  |
| *OsHAK21.a* | GGTGGAGGTCGTCGTCT | TGGCTTCATCGGTTCA |  |
| *OsHAK21.b* | ATGAGTCTGGCGTTCC | CGATCCATTTAATTTGGT |  |
| *OsNHX1.a* | GATAAATCACGAGCAAA | TTAACAATGCAGGCTAT |  |
| *OsABCI7.a* | CGTGGATTCAGGGAGG | AGATGGAGGATAAGAGGGA |  |

**Table S3:** Twenty-nine *japonica* rice germplasm accessions with different salt tolerances, names, source, and STS

| **No.** | **Name** | **Origin** | **STS** | **No.** | **Name** | **Origin** | **STS** |
| --- | --- | --- | --- | --- | --- | --- | --- |
| 1 | Bertone | Portugal | 7.5 | 16 | ^4154-4 | Jiangsu, China | 3.3 |
| 2 | Agostono | Italy | 7.2 | 17 | Huayu 2 | Ningxia, China | 3.0 |
| 3 | Huangluo | Russia | 6.6 | 18 | Nipponbare | Japan | 2.9 |
| 4 | Nongke 843 | Ningxia, China | 6.5 | 19 | Banat 725 | Australia | 2.6 |
| 5 | Changbai 9 | Jilin, China | 6.5 | 20 | Liaokai 79 | Liaoning, China | 2.6 |
| 6 | Banat 2951 | Australia | 6.5 | 21 | Yuncundao | Korea | 2.4 |
| 7 | Huinuo | Japan | 6.5 | 22 | Jianan 8 | Taiwan, China | 2.2 |
| 8 | Cigalon | France | 6.5 | 23 | Longjing 22 | Heilongjiang, China | 2.2 |
| 9 | Xindao 10 | Xinjiang, China | 6.4 | 24 | Qinglin 518 | Jilin, China | 2.2 |
| 10 | Xiaobaibandao | Ningxia, China | 6.4 | 25 | Xinzhu 8 | Taiwan, China | 2.1 |
| 11 | Yangbiguangkeludao | Yunnan, China | 6.3 | 26 | Koshihikari | Japan | 1.8 |
| 12 | Chenggonghangu | Yunnan, China | 6.2 | 27 | Xiannan 22 | Korea | 1.7 |
| 13 | Zhongkechang 6 | Beijing, China | 6.0 | 28 | Shanfuliya | Guinea | 1.4 |
| 14 | Yanghebaipidao | Ningxia, China | 5.8 | 29 | Qianchonglang | Japan | 1.4 |
| 15 | Ningzi 629 | Ningxia, China | 4.1 |  |  |  |  |

## Supplementary Figures


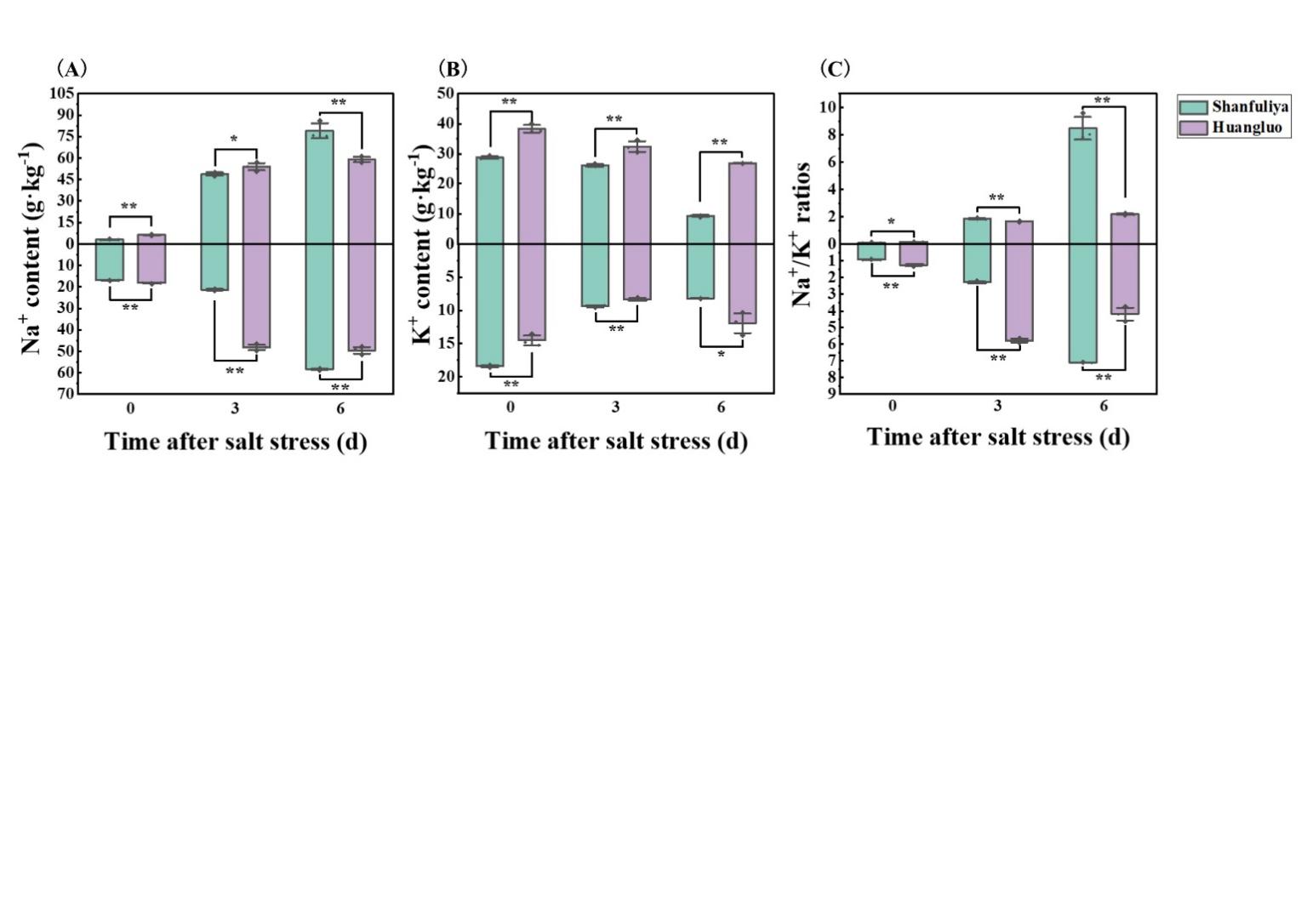


**Figure S1.** Ion content and Na^+^/K^+^ ratios of *japonica* rice germplasm with different salt tolerances at different times. (A) Na^+^ content; (B) K^+^ content; and (C) Na^+^/K^+^ ratios. Relative levels in the rice shoot and root are shown at the top and bottom of the X-axis, respectively. * p < 0.05, ** p < 0.01.


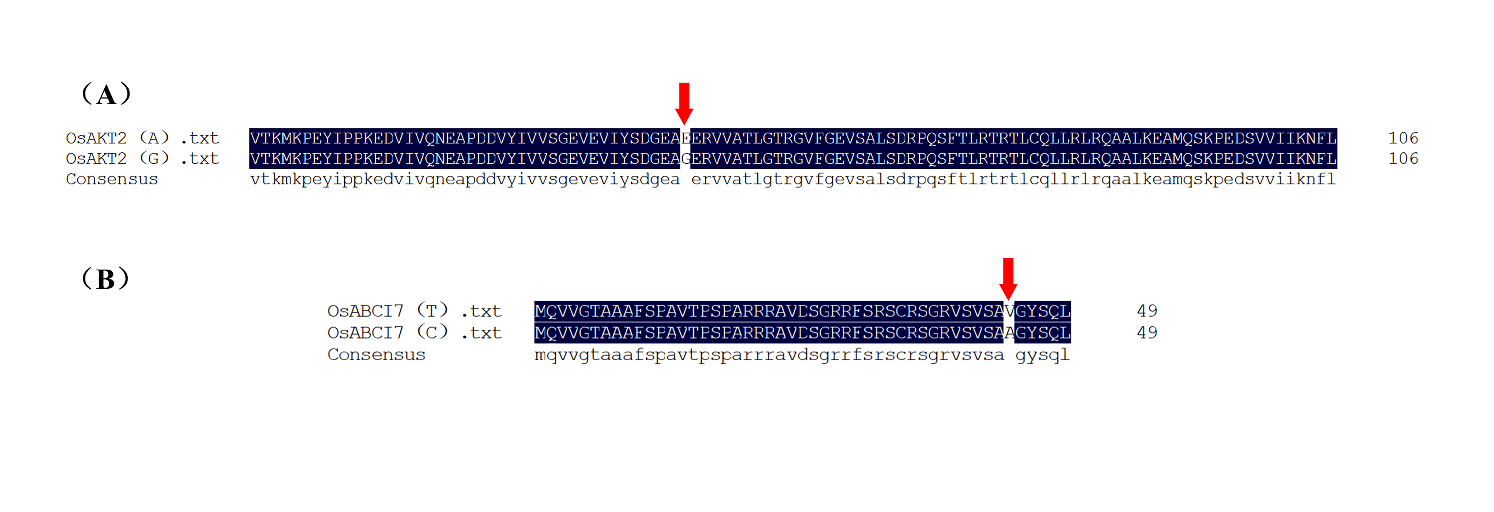


**Figure S2.** Amino acid substitutions in OsAKT2 and OsABCI7; red arrow shows site with different amino acid.
